# A proteome scale study reveals how plastic surfaces and agitation promote protein aggregation

**DOI:** 10.1038/s41598-023-28412-7

**Published:** 2023-01-21

**Authors:** Marion Schvartz, Florent Saudrais, Stéphanie Devineau, Jean-Christophe Aude, Stéphane Chédin, Céline Henry, Aarón Millán-Oropeza, Thomas Perrault, Laura Pieri, Serge Pin, Yves Boulard, Guillaume Brotons, Jean-Philippe Renault

**Affiliations:** 1 Université Paris-Saclay, CEA, CNRS, NIMBE, LIONS, 91191 Gif-Sur-Yvette, France; 2 Institut des Molécules et Matériaux du Mans (IMMM), UMR 6283 CNRS, Le Mans Université, Avenue Olivier Messiaen, 72085 Le Mans Cedex, France; 3 Université Paris Cité, CNRS, Unité de Biologie Fonctionnelle et Adaptative, 75013 Paris, France; 4 Université Paris-Saclay, CEA, CNRS, Institute for Integrative Biology of the Cell (I2BC), 91198 Gif-Sur-Yvette, France; 5 Université Paris-Saclay, INRAE, AgroParisTech, Micalis Institute, PAPPSO, 78350 Jouy-en-Josas, France

**Keywords:** Biomaterials - proteins, Colloids, Biomedical engineering

## Abstract

Protein aggregation in biotherapeutics can reduce their activity and effectiveness. It may also promote immune reactions responsible for severe adverse effects. The impact of plastic materials on protein destabilization is not totally understood. Here, we propose to deconvolve the effects of material surface, air/liquid interface, and agitation to decipher their respective role in protein destabilization and aggregation. We analyzed the effect of polypropylene, TEFLON, glass and LOBIND surfaces on the stability of purified proteins (bovine serum albumin, hemoglobin and α-synuclein) and on a cell extract composed of 6000 soluble proteins during agitation (*P* = 0.1–1.2 W/kg). Proteomic analysis revealed that chaperonins, intrinsically disordered proteins and ribosomes were more sensitive to the combined effects of material surfaces and agitation while small metabolic oligomers could be protected in the same conditions. Protein loss observations coupled to Raman microscopy, dynamic light scattering and proteomic allowed us to propose a mechanistic model of protein destabilization by plastics. Our results suggest that protein loss is not primarily due to the nucleation of small aggregates in solution, but to the destabilization of proteins exposed to material surfaces and their subsequent aggregation at the sheared air/liquid interface, an effect that cannot be prevented by using LOBIND tubes. A guidance can be established on how to minimize these adverse effects. Remove one of the components of this combined stress - material, air (even partially), or agitation - and proteins will be preserved.

## Introduction

The number of protein-based therapeutics is increasing rapidly. However, proteins are exposed to stresses during manufacturing, affecting their stability. The aggregation of proteins in biotherapeutics can reduce their activity and effectiveness, but it may also promote immune reactions responsible for severe adverse effects such as allergic responses and anaphylaxis^1^.

Various strategies have been developed to avoid protein aggregation during biotherapeutics processing either by carefully controlling the local environment and process or by removing aggregates before sampling. The physical and chemical factors that determine the stability of proteins or trigger their aggregation have long been the subject of research in the pharmaceutical and biochemical fields. Agitation, temperature, pH and ionic strength are known to induce protein aggregation^1–3^.

The effect of materials on protein stability in biotherapeutics processing has been comparatively less studied. Indeed, the current picture has long been one of minimal loss of proteins by adsorption on surfaces, where protein/material interactions would only result in surface passivation without affecting the remaining free proteins in solution. From the 1970s, Leo Vroman^4,5^ demonstrated that protein adsorption was not a static process, but rather a dynamic one where exchanges occurred at the liquid–solid interface between free and adsorbed proteins depending on the protein concentration and affinity for the surface. This model can now be completed by the partial loss of protein stability following weak interactions with the surface and subsequent release in the solution^6^, a first step to a remote effect of interfaces.

Recent studies have demonstrated that the destabilizing effect of solid/liquid interfaces on protein stability was more profound than initially believed, and could be enhanced by stirring. Synergistic effects of flow and surfaces on antibody aggregation have already been described^7,8^. Other studies emphasized the role of air bubbles^9,10^. These observations suggest that both the solid/liquid, the air/liquid interface and agitation are key players when considering the effect of a novel process or material during biotherapeutics production. However, the mechanistic description that could explain the processes involved and the interplay between the different interfaces and agitation on protein stability is missing. Moreover, most experimental studies were conducted with single purified proteins, which cannot account for the role of protein–protein interactions when different proteins are involved, the possible association and dissociation of protein complexes at interfaces^11^, and the variations in protein sensitivity to these stresses.

Here, we propose to deconvolve the effects of the different players - material surface, air/liquid interface, agitation - to decipher their role in protein aggregation. We extended this analysis from purified model proteins to a cellular extract composed of thousands of different proteins to identify the most sensitive proteins to this type of combined stresses.

In this study, we analyzed the surface-induced destabilization of purified proteins and a complex mixture of soluble proteins by contact with plastic surfaces in aqueous solution. Polypropylene (PP), polyfluoreethylene (PTFE or TEFLON), and a reference borosilicate glass vial, which are commonly used materials for drug packaging and protein manipulations, were chosen to investigate the combined effect of surfaces and agitation on protein aggregation. We also included Protein LOBIND tubes (EPPENDORF), which are designed to reduce protein adsorption on surfaces, in order to compare the effects observed with other plastics to this material.

In the first instance, we analyzed the effects of plastic surface and agitation on three different purified proteins. We chose bovine serum albumin (BSA), porcine hemoglobin (Hb) and human α-synuclein (α-syn) because of their differences in structure and thermal stability (see sup inf. Table S1 and S2). Hb is a tetrameric hemoprotein located in erythrocytes that binds oxygen. It is classified as ‘hard proteins’ according to Norde’s definition of protein-surface interactions due to its relatively high stability^12^. BSA is a monomeric circulating protein that transports different types of biomolecules and drugs in the blood stream and that can be classified as a “soft protein“ owing to its capability to rearrange its conformation upon adsorption^13^. α-syn was chosen for its tendency to aggregate. The aggregation of α-syn has been associated with Parkinson neurodegenerative disease^14^. It is an intrinsically disordered protein (IDP) that is partially unfolded in its native state. Then, we investigated protein aggregation with a cell extract (YPE, Yeast Protein Extract). Soluble proteins were extracted from *Saccharomyces cerevisiae*. YPE is built from thousands of soluble proteins with a high dynamic range and has been fully characterized by proteomics^15–17^.

The protein solutions were mixed at 6 °C in tubes made of each of these 4 materials attached to a rotating wheel to monitor the protein loss as a function of the mechanical energy and power per solution mass. The enhanced gravitationally driven agitation was chosen to quantify the amount of energy supplied to the sample precisely. The power, expressed in watts per fluid mass unit, ranged from 0.12 W/kg to 1.2 W/kg. This experimental design allows us to apply the Kolmogorov principles ^18,19^ to quantify the shear stress in solution, while varying the volume of air and liquid in the samples. These conditions mimic moderate stress (low temperature, neutral pH, physiological ionic strength) in the presence of two different interfaces (air/liquid and solid/liquid) with agitation, conditions that are frequently used for protein preparation and handling.

## Results and discussion

### Observation of protein loss in purified protein solutions

**Figure 1:**
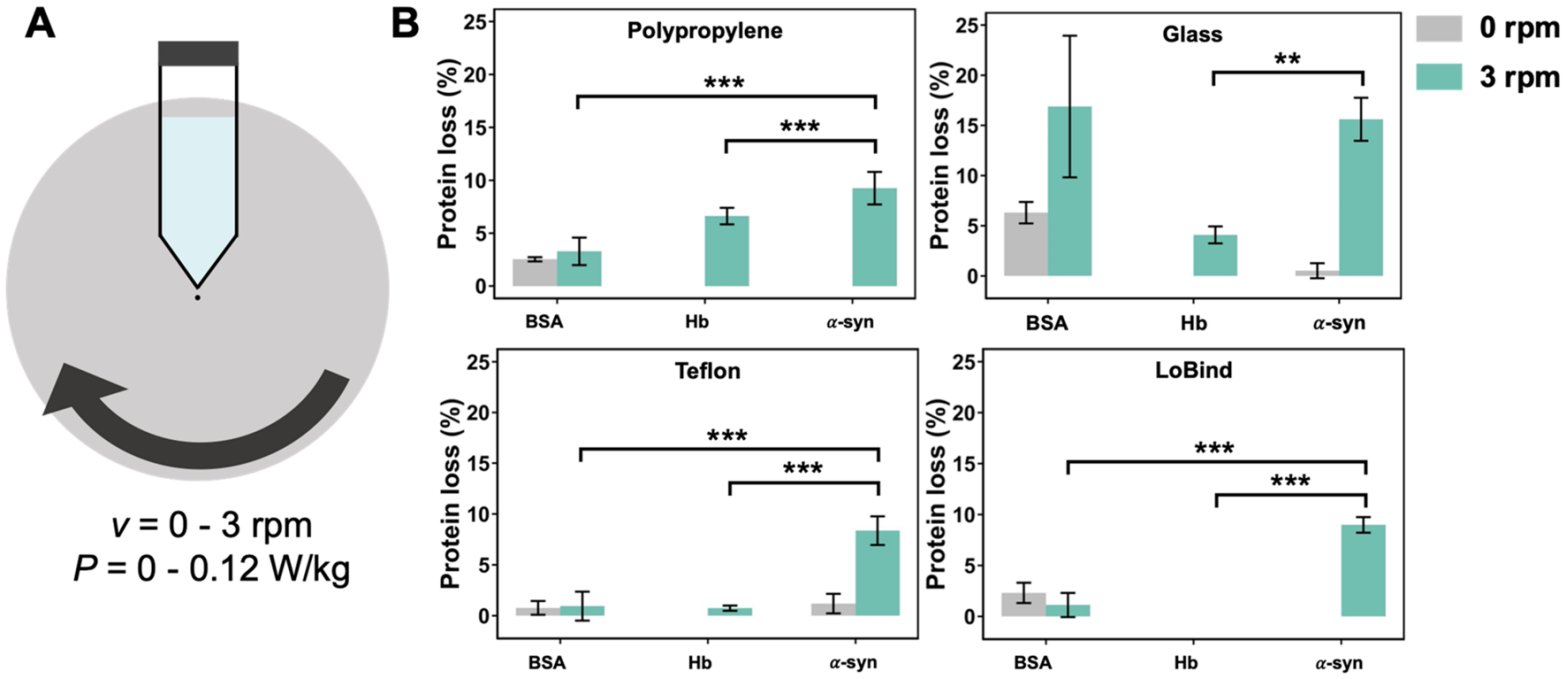
(**A**) Scheme of the experimental setup designed to measure protein loss during agitation of purified protein solutions with a rotating wheel. The speed ranged from 0 to 3 rpm, corresponding to a power from 0 to 0.12 W/kg. Tubes made of PP, glass, TEFLON, LOBIND were used. A conical PP FISHERBRAND tube is represented. (**B**) Protein loss of BSA, Hb, α-syn solutions measured in PP, glass, TEFLON and LOBIND tubes after 24 h at 6 °C without (grey) and with agitation at 3 rpm (green) on a rotating wheel. The initial protein concentration was C_0_ = 0.1 g/L (V = 10 mL). Significant differences are determined using the Tukey’s “honestly significant difference” test realized from variance analysis (Anova) (*** *p*-value < 0.001; ** *p*-value < 0.01). To observe the effect of the protein nature, only the differences at 3 rpm are shown, all the statistical results are available in Table S3.

The experimental set-up designed to monitor the protein loss during agitation of the protein solution in PP, glass, TEFLON and LOBIND tubes with a rotating wheel is represented in Fig. 1A. All the experiments were conducted in 100 mM phosphate buffer pH 7 at 6 °C. First, we measured the protein loss of purified proteins as a function of the tube material with and without agitation at 3 rpm (Fig. 1B). The protein concentration was measured after agitation to calculate the corresponding protein loss. Fluorescence and UV–vis spectroscopy were used to measure the initial and final concentrations of BSA, Hb, and α-syn respectively.

The protein loss for Hb and α-syn solutions is triggered by agitation for all the tested materials, except for LOBIND tubes. Indeed, no Hb loss was measured (within the detection limit) without agitation for PP, TEFLON, and glass, while a Hb loss up to 7% was measured with PP under agitation. Similarly, little or no α-syn loss was observed for all the materials (< 1%) without agitation while it increases to 9% with PP, TEFLON and LOBIND, and to 16% with glass. The different levels of protein loss suggest that α-syn is more sensitive to destabilization during moderate agitation compared to Hb, and that this effect can be enhanced depending on the material.

A different trend is observed for BSA as the protein loss remains similar with and without agitation for PP (3%), TEFLON (< 1%), and LOBIND (1–2%) tubes. On the contrary, a dramatic effect of agitation is observed when BSA solutions are in contact with glass, with a BSA loss increasing from 6 to 17% with agitation.

Overall, the protein losses observed here for purified proteins with agitation are both protein- and surface-dependent. The level of protein loss can be ranked as BSA < Hb < α-syn for PP, while different trends are observed for the other surfaces. Because each material exhibits a different effect in terms of protein destabilization during agitation depending on the protein nature, it is thus necessary to include a larger set of proteins with various physical chemical and structural features to gain a more realistic and global understanding of this process.

### Observation of protein loss in cell extracts

**Figure 2:**
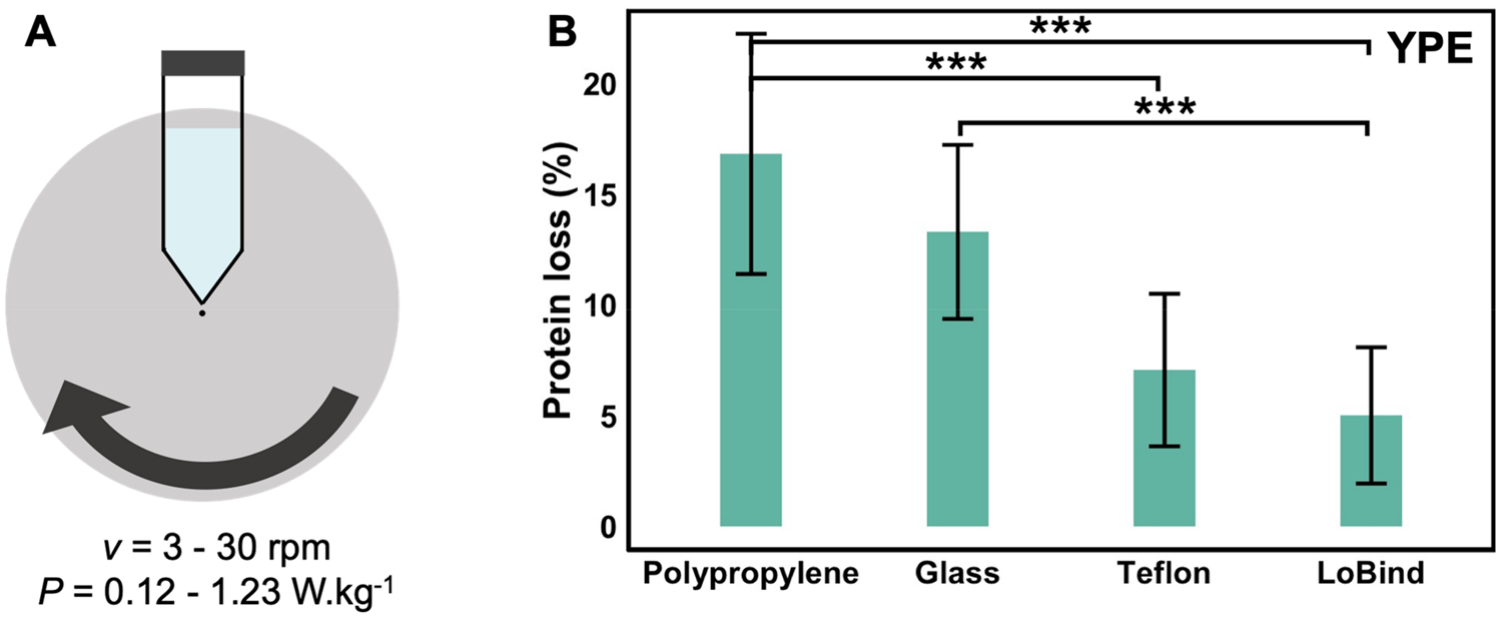
(**A**) Scheme of the experimental setup designed to measure protein loss during agitation of a protein solution with a rotating wheel. The speed ranged from 3 to 30 rpm corresponding to a power of 0.12 to 1.23 W/kg. Tubes made of PP, glass, TEFLON, LOBIND were used. A conical PP FISHERBRAND tube is represented. (**B**) Protein losses measured after 24 h agitation of YPE cell extract at 6 °C and 3 rpm in each tube. The initial protein concentration was C_0_ = 0.1 g/L and filled volume 60%. The error corresponds to the standard deviation for three biological replicates. Significant differences are determined using the Tukey’s “honestly significant difference” test realized from variance analysis (Anova) (*** *p*-value < 0.001).

Second, we measured the protein loss for a complex mixture of thousands of soluble proteins using yeast protein cell extract (YPE). The protein loss was measured after moderate agitation of the protein solution for 24 h at 6 °C in PP, glass, TEFLON, and LOBIND tubes on a rotating wheel at a speed of 3 to 30 rpm. The YPE was treated with a cocktail of protease inhibitors to prevent enzymatic protein degradation. The initial and final protein concentration was measured using UV spectroscopy and the percentage of lost protein calculated (Fig. 2).

The maximum protein losses were observed for PP and glass tubes. A significant protein loss was also observed in TEFLON and LOBIND tubes. Higher levels of protein loss were measured for YPE compared to Hb, BSA and α-syn on PP. Significant protein losses were observed in all agitation conditions to a level that cannot only be due to protein adsorption on the material surfaces. Indeed, the maximum losses measured in glass vials correspond to ten times the higher mass reported by our group for covering silica surfaces using the same YPE protein extract solution^17^. Furthermore, the protein losses were comparable between LOBIND plastic (7 ± 3%) expected to be non-adsorbing for proteins^20,21^, and PTFE (5 ± 3%) expected to be a strong binder because of hydrophobic interactions ^22,23^. Measured losses did not simply follow the values of the solutions/material’s interfacial tensions - estimated via contact angle measurements presented in Figure S1 - and did not happen in the absence of agitation (within experimental errors, Table S4). All the observations suggest that the protein loss is related to the presence of solid/liquid (S/L) and air/liquid (A/L) interfaces, and are thus more complex than a simple adsorption phenomenon on the material surface.

**Figure 3:**
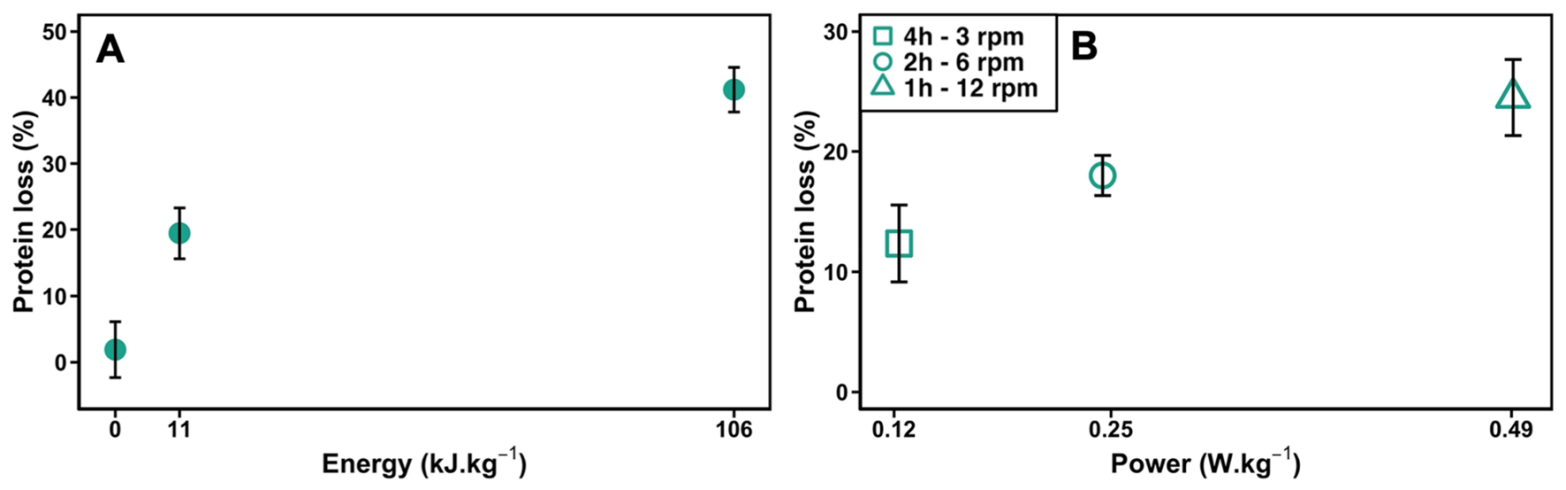
Evolution of protein loss for YPE in PP tubes under agitation as a function of (**A**) the energy supplied to the system for a fixed duration of 24 h and a fixed power of 1.23 W/kg, (**B**) the power applied for a fixed energy of 1.8 kJ/kg. All samples were mixed at 6 °C on a rotating wheel.

Because PP induced the larger protein loss, we focused our experimental work on this largely used material to understand the processes involved in surface-induced protein degradation under agitation. First, we varied the energy injected in the system by increasing the wheel rotation speed for a fixed duration of 24 h (Fig. 2A). The protein loss increased with the energy supplied to the system, reaching values as high as 45% when an energy of 106 kJ/kg (corresponding to 30 rpm) was applied. We also monitored the protein loss as a function of the power applied at a fixed energy by varying the rotating duration. The protein loss doubled for a fixed received energy of 1.8 kJ/kg when the solution was mixed for 4 h at 3 rpm compared to 1 h at 12 rpm corresponding to a power of 0.123 and 0.492 W/kg respectively (Fig. 3).

The increase of the protein loss with the energy supplied points to a synergistic effect of the contact with PP and agitation on surface-induced protein aggregation. This observation was already reported for other types of materials^2^. However, the dependence of the protein loss on the power applied at fixed energy strongly suggests that turbulence plays a role in the process.

**Figure 4:**
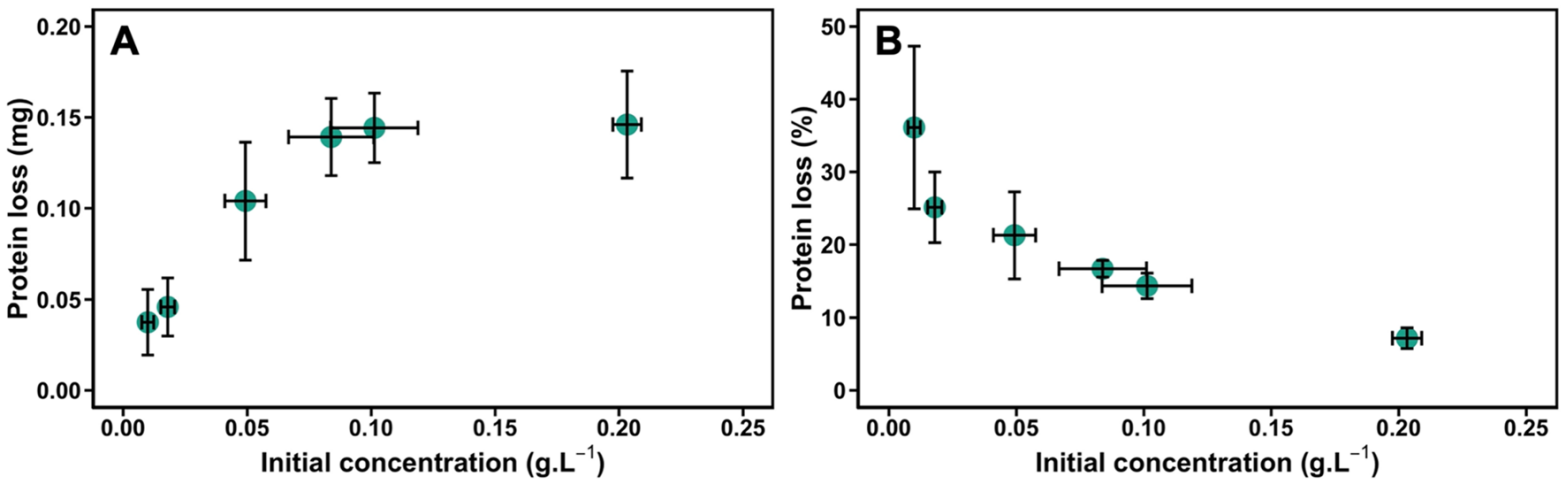
Effect of the initial protein concentration on the protein loss for YPE cell extract. (**A**) Absolute protein loss in mg. (**B**) Percentage of protein loss. Samples were gently mixed in PP tubes on a rotating wheel at 3 rpm during 4 h at 6 °C.

The initial concentration of the protein solution also affects the protein loss due to surface-induced aggregation (Fig. 4). It increased from 0.04 to 0.14 mg (3.5-fold increase) when the protein concentration was multiplied by 10 from 10 mg/L to 100 mg/L (Fig. 4). The saturation plateau corresponding to a maximum protein loss of 0.15 mg observed at the highest initial protein concentrations recalls adsorption isotherms and highlights the role of interfaces in the process.

### Critical interfaces for the protein loss

**Figure 5:**
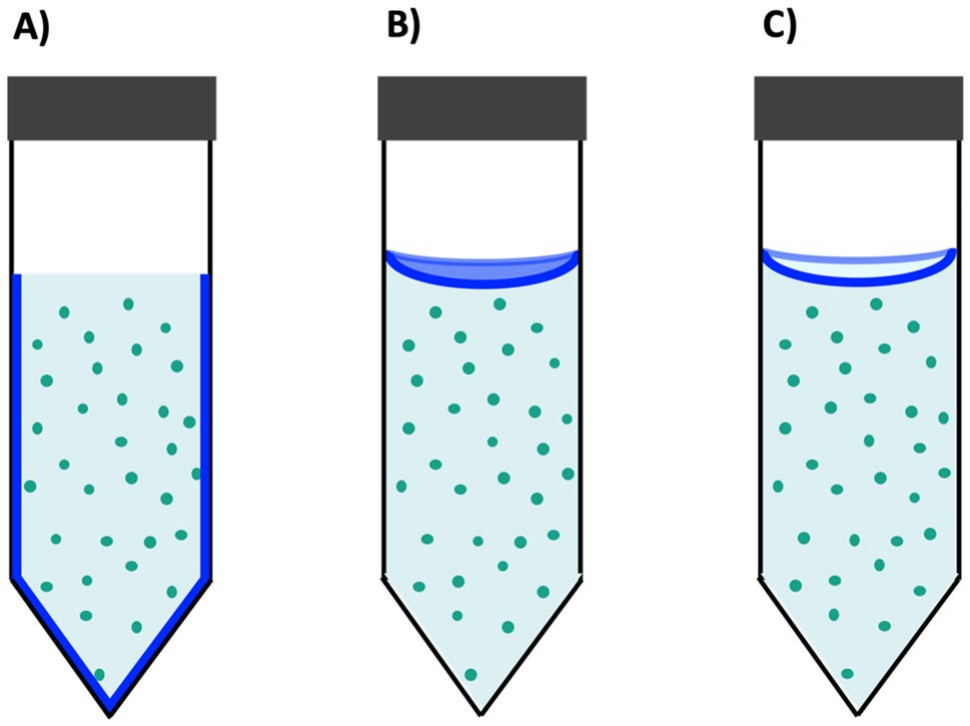
Scheme picturing the three interfaces considered during protein agitation. (**A**) Solid/liquid interface at the material surface. (**B**) Air/liquid interface. (**C**) Triple air/liquid/solid line interface defining the meniscus. Proteins, represented as green dots, could adsorb to one or several of these interfaces during mixing.

Proteins could adsorb to three different types of interfaces in the experiments: the solid/liquid (S/L) interface, the air/liquid (A/L) interface at the top of the solution, and the triple air/solid/liquid (A/L/S) line interface at the meniscus, all of which are continuously renewed during mixing (Fig. 5). The specific surface area of each of these interfaces in our system is evaluated, depending on the tube orientation, to 5–45 cm^2^ for the S/L interface, 1–14 cm^2^ for the A/L interface (at rest), and 4–23 cm for the A/L/S interface, depending on the volume of solution and the tube orientation (vertical or horizontal) (Table 1).

**Table 1:** Protein loss (%) for YPE cell extract in PP tubes as a function of the volume of solution and the evolution of the amount of A/L, S/L and A/S/L interfaces during the agitation (excluding waves and bubbles).

V_solution_ (mL)	V_air_ (mL)	Protein loss (%)	A/L (cm^2^)	S/L (cm^2^)	A/L/S (cm)
1	15	14.9 ± 2.5	1 ↔ 4	~ 5	~ 4
3	13	13.5 ± 2.4	2 ↔ 12	~ 11	5 ↔ 20
10	6	14.7 ± 3.8	2 ↔ 14	~ 31	5 ↔ 23
12.5	3.5	0 (− 0.73 ± 1.3)	2 ↔ 13	~ 39	5 ↔ 23
15	1	0 (− 5 ± 0.8)	2 ↔ 5	~ 45	5 ↔ 8

The protein loss referred to a solution agitated at 3 rpm for 24 h at 6 °C (C_0_ = 0.1 g/L).

By varying the amount of solution in the tubes (and the corresponding volume of air), we compared the relative effect of each of these interfaces for different surface-to-volume ratios (Fig. 5, Scheme S1). Reducing the amount of air, thus increasing the ratio of S/L interface in comparison to others, reduce the protein loss. On the other hand, we observed that the S/L interface renewal leads to a higher protein loss (Figure S3). It suggests that S/L interface, once passivated by proteins, has a minor effect on proteins destabilization in solution (Table 1) and that A/L and/or A/L/S are the determining interfaces. Different studies ^24^ suggest that the A/L/S moving triple line defines a zone of complex drying phenomena that can induce strong laterals forces^25^ and ‘shear’ damage to proteins^3^. To test this hypothesis, we changed the geometry of the system and used a dip coater setup with a PP plate that was vertically immersed and withdrawn in order to reproduce the condition encountered in the rotating wheel agitation experiments at the triple A/L/S line interface (Scheme S2). We neglected the difference of PP surface curvature in both geometries, but we kept a motion period that reproduces the conditions of the rotating wheel set-up. No significant protein loss was observed in the dip-coating experiments (Table S5), contrary to the observations reported in the case of insulin solutions^24^. Therefore, drying at the triple line zone with PP is certainly not the process responsible for the protein loss measured in the rotating wheel experiment.

We can thus reasonably assume a minor role of the S/L and A/L/S interfaces in the protein loss process for wheel rotating agitation in contact with PP. Therefore, we expect that the critical interface is probably the A/L one. Note that the A/L interface deformations in the dip coating experiment does not seem appropriate for generating stresses that aggregate proteins at a significant level (Scheme S2). Furthermore, very little protein loss was observed upon agitation in tubes above a given volume of solution (approximately 75% of the total volume) (Table 1). On the contrary, a major protein loss was measured in all conditions for V_solution_ < 60%. Hence, the transition from protein aggregation to protein stabilization, *i.e.* from stressful to stress-free agitation conditions, seems rather steep and strongly depends on the ratio V_solution_ / V_air_.

Based on the observation of the tubes during wheel rotating agitation (Figure S6), we could describe this boundary qualitatively as the transition between a ‘gas moving into the liquid’ to a ‘liquid falling into the gas’ status. This is sometimes described as the transition from plug flow, where steady elongated bubbles are formed, to wavy stratified flow^26^. (Figure S7) The change in the flow pattern is associated with the apparition of fluctuations in the amount of A/L interface due to the formation of waves or bubbles. It is equivalent to repetitive compression/decompression events at interfaces, which are known to be highly detrimental to proteins^9,27^. This is probably the key mechanism at play here.

We must also discuss the possible interplay of turbulences (Fig. 3) and of the surface nature of the material (Fig. 2). We must first notice that the envisioned turbulent shear forces (Table S7) are much smaller than the forces required to unfold proteins, irrespective of the protein size. The shear forces in the system are in the fN range (Table S7), whereas tens to hundreds of pN are required to unfold proteins^28^. We are considering here a gentle agitation process. However, the shearing, even if it cannot alter the protein directly, can have two effects: (i) it can increase and decrease transiently the amount of A/L interface (the wavy behaviour^29^) thus favouring the aggregation at A/L; (ii) it may favour the desorption of adsorbed native or destabilized proteins from the S/L or A/L interfaces^24,30^.

The role of the surface material nature can operate either directly through the adsorption of native proteins, leading to the formation of nucleation sites for aggregation, or through the destabilization of protein in solution, by a favourable interaction with protein unfolded/destabilized conformations^6,31^.

We cannot say from these data if the effect of turbulences precedes, succeeds or acts in parallel to the effect of the material. To clarify this, we conducted an additional experiment involving an excess surface of plastic, in the form of a porous PP filter with a specific surface area of 0.7 m^2^/g (Scheme S3 and Figure S2). It constitutes a high plastic surface exposed to the protein solution in absence of turbulences. With the obtained S/L interface of 120 cm^2^ during agitation (compared to 50 cm^2^ during agitation without filter), the protein loss from YPE doubled and reached 40% of the initial concentration. (Table S6).

Along with Figure S3, this suggests that the material surface initiates some protein destabilization that is potentiated by the combination of A/L interface and turbulence. The thermodynamic force is not the interaction between the material and the protein in their native state (nucleation model from Sluzky^32^), but the strong interaction between the material and the protein in their unfolded/destabilized conformations that shifts all folding equilibria in solution. ^6^ This situation is similar to other stresses, like pressure and freezing, that have been described to induce such conformational drifts ^33^ by microscopic cycles of protein–protein associations/dissociations. Therefore, this mechanistic model also explains why surfaces enclosed with low amounts of A/L interfaces, such as the plastic filter presented here, or designed to limit protein adsorption, like LOBIND plastic, can also trigger significant destabilizing effects on proteins in solution.

### Fate of lost proteins

**Figure 6:**
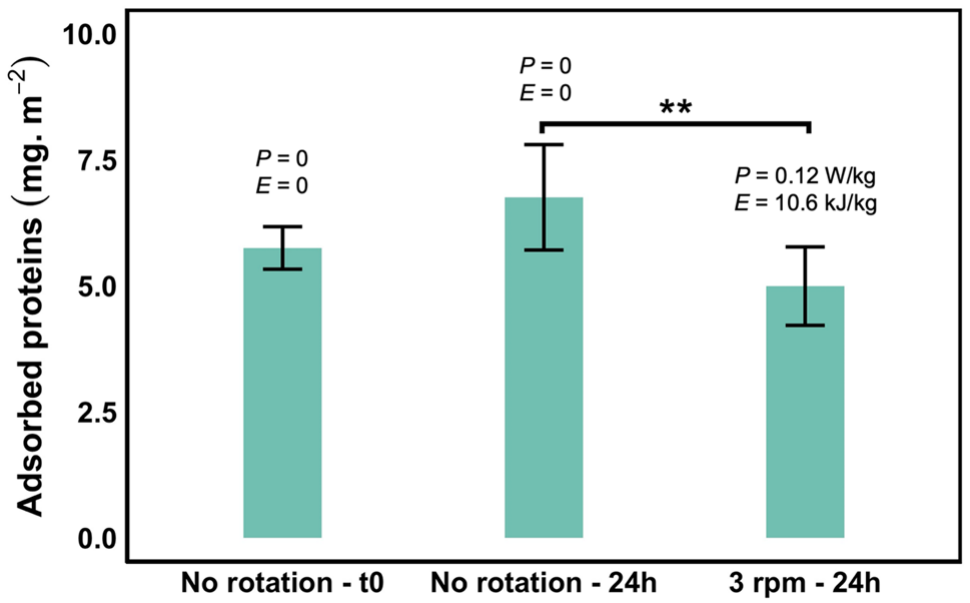
Amount of adsorbed proteins on PP tubes in mg/m^2^ as a function of the rotational speed for YPE cell extracts. The power (P) and energy (E) are indicated for each condition. The adsorbed proteins were removed using 0.1% v/v sodium dodecyl sulfate (SDS) and the amount of proteins measured by UV spectroscopy. Significant differences are determined using the Tukey “honestly significant difference” test realized from variance analysis (Anova) (** *p*-value < 0.01).

A major question is what happens to the lost proteins. First, we determined whether the protein loss could be explained by proteins being trapped on the material surface. In this case, the thickness of the adsorbed protein layer should increase with the number of cycles to reach the measured protein loss. The adsorbed proteins on PP tubes were removed using 0.1% v/v sodium dodecyl sulfate (SDS), a strong surfactant, and the corresponding protein amount measured by UV spectroscopy. The mass of adsorbed proteins on PP (in mg/m^2^) did not significantly increase as a function of the rotational speed (Fig. 6). It even decreased. It also represents a relatively small amount (25 µg) compared to the total protein loss of 150 µg, that is < 20% of the total protein loss. Therefore, it is unlikely that protein adsorption on the material surface alone is responsible for the protein loss. Thus, we focused our study on newly formed objects in solution to understand the fate of lost proteins.

**Figure 7:**
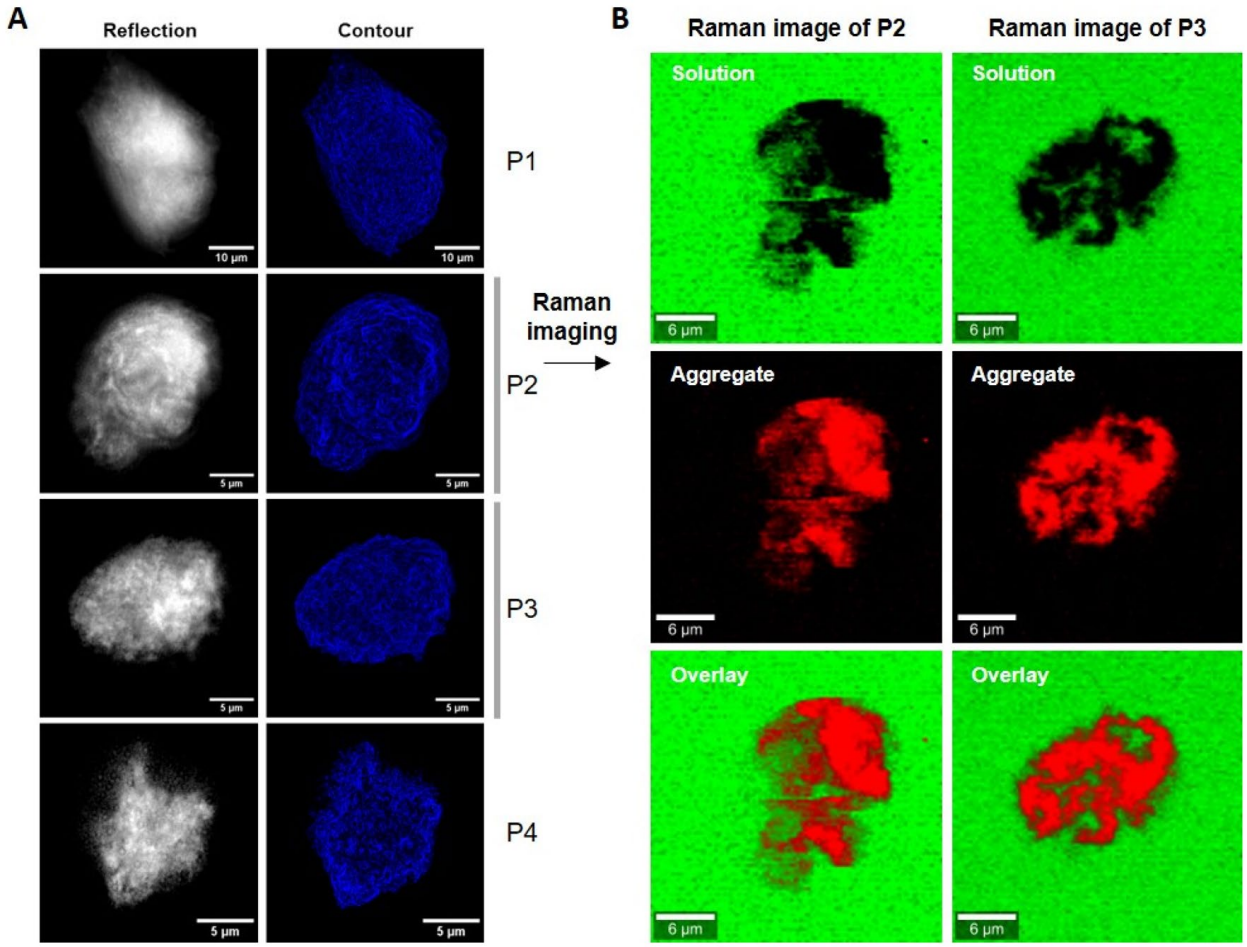
In situ imaging in solution and biochemical analysis of the protein aggregates. (**A**) Optical images using reflected light and contour reconstruction. (**B**) Raman images of the particles P2, P3. Images corresponding to the Raman spectra of the protein aggregate and solution are shown in red and green, respectively. The overlay is shown at the bottom. Two biological replicates were analyzed for each condition.

Large particles (d > 1 µm) were identified in the protein solutions after wheel rotating agitation in PP tubes. Optical microscopy using reflected light and contour reconstruction with Fiji software were applied to better visualize their shape and structure (Fig. 7A). The size of the objects ranged from a few to tens of micrometers, though no inner structure could be seen.

Their biochemical composition was analyzed by confocal Raman microscopy (Fig. 7B). Raman characteristics (see attributions in table S8) confirmed the protein nature of the particles, in agreement with the observed protein loss, with the possible presence of DNA or RNA, suggesting that ribosomes/nucleoproteins might be included in the particles. These data suggest that protein aggregation is indeed responsible for the protein loss, as reported before in the case of purified immunoglobulins submitted to periodic compression decompression^27,34,35^.

**Figure 8:**
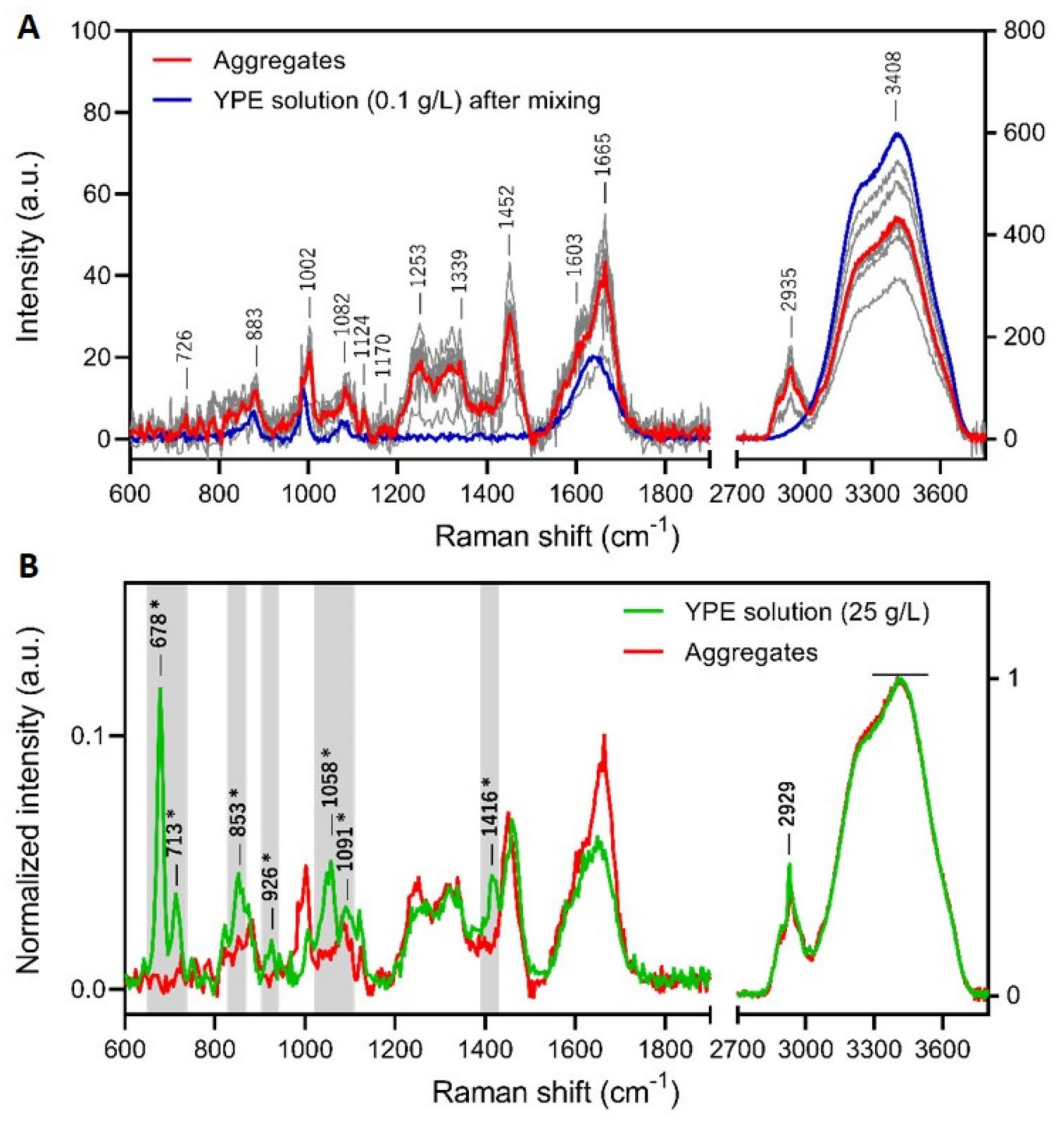
Raman spectra of protein aggregates and YPE solution. (**A**) Average Raman spectra of aggregates formed in YPE solution at 0.1 g/L (red) and YPE solution at 0.1 g/L after mixing (blue). The individual spectra of protein aggregates are shown in grey. (**B**) Normalized Raman spectra of protein aggregates formed in YPE solution at 0.1 g/L (red) and YPE stock solution at 25 g/L (green). The spectra were normalized to the OH band of water at 3406 cm^−1^. * The Raman bands that are present in YPE solution but absent in protein aggregates are highlighted in grey.

Raman imaging revealed also a slightly different pattern compared to bright field images with patches of denser protein cores and inlets of solution in the particles. In order to distinguish between protein gelation and aggregation, we determined the level of hydration of the protein particles by confocal Raman microscopy. The Raman spectra of the particles compared to the surrounding YPE solution after mixing are shown in Fig. 8A. The water content of the sample is reflected by the intensity of the OH stretching vibration band of water around 3400 cm^−1^. Though it can be difficult to compare the absolute intensity of Raman bands, the spectra were taken here in the same experimental conditions and within the same sample. The average intensity of the OH vibration band of the protein aggregates is only 30% lower than the one of the surrounding solution. Thus, the protein particles that formed during mixing in PP tubes retained a significant water content, suggesting that they are more gel-like protein particles than dehydrated and denatured protein compact aggregates.

We compared also the Raman spectra of the particles formed to the one of the initial YPE solution (Fig. 8B). However, we had to work with solutions at a higher concentration (25 g/L) in order to have a good signal to noise ratio for free proteins (Figure S8). The spectra of the aggregates and the initial solution show major differences, highlighted in grey in the figure. Several bands well visible in YPE solution are missing from the spectrum of the particles, showing that some cofactors are excluded from the aggregation process (see table S9), an effect that could also be favored by protein structural changes during aggregation. By contrast, little difference was observed between the Raman spectra of various protein aggregates apart from their water content, suggesting that the particles formed in this size range are relatively homogeneous in composition (Fig. 8A).

**Figure 9:**
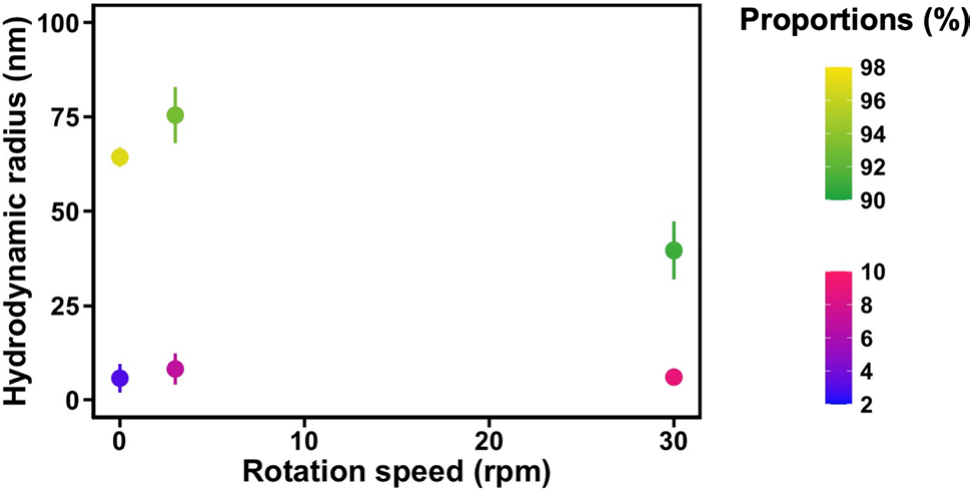
Analysis of the hydrodynamic radius of particles formed in YPE solution after mixing in PP tubes on a rotating wheel as a function of the speed. The intensity size distribution was measured by DLS after filtration of the solutions at 1.2 µm to remove the larger aggregates. The percentage of each population (in intensity) is coded in color.

In a second step, we searched for potential intermediates of particle growth. The solutions were filtered at 1.2 µm to remove large aggregates and analyzed by Dynamic Light Scattering (DLS) after 24 h with or without mixing. The hydrodynamic radius of the objects as a function of the rotational speed is shown in Fig. 9. Interestingly, two distinct populations with an average diameter of 10 and 130 nm were identified in steady conditions without mixing. Since the cellular extract is composed of thousands of different proteins, we can assume in the first place that these two populations correspond to free proteins and large protein complexes respectively. This distribution in two population may seem at first surprising. Indeed simulation at the proteome scale of protein diffusive properties (which is indeed the physical parameters measured by DLS) does not predict two population^36^. However, one must keep in mind that large transcription complexes are very abundant in rapidly dividing cells where they may represent up to one third of the protein content^37^. Besides ribosomes, recent mass spectrometry data suggest that two third of the protein exist as “separable complexes” in eukaryotic cell extracts^38^. Our observation may simply reflect these facts.

At 3 rpm, we observed a small increase in the hydrodynamic radius of the larger objects that represent 93% of the particles in intensity. At 30 rpm, both the size and the number of the larger objects decreased, while the size of small objects did not change significantly. These results suggest that no aggregates with a diameter 100 nm < d < 1 µm formed in the solution.

The lack of detectable aggregate of intermediate size in all the conditions tested (variation of solution volume: 0, 10, 15 mL and mixing duration (up to 53 h) is surprising. It suggests that protein aggregation does not rely a progressive growth in solution. Instead, large protein particles seem to form directly during agitation. We further verified that no particle nucleation/growth was occurring inside solution using a seeding experiment (see supporting information Figure S5). The introduction in fresh cell extracts of already aged solution, either filtered to remove particle, or unfiltered, did not indeed enhance the protein degradation.

Therefore, we suggest that the particles are formed through a wrinkling/wrapping process from the protein layers of the A/L interface. The size of the particles is comparable with the calculated Kolmogorov length scale^39^ (Table S7) that links the maximum shear rate and dimension of particles. Therefore, the agglomeration process that takes place at a macroscopic interface would lead to micron size peeled-off protein aggregates through shearing and turbulences.

### Mechanistic proposition

**Figure 10:**
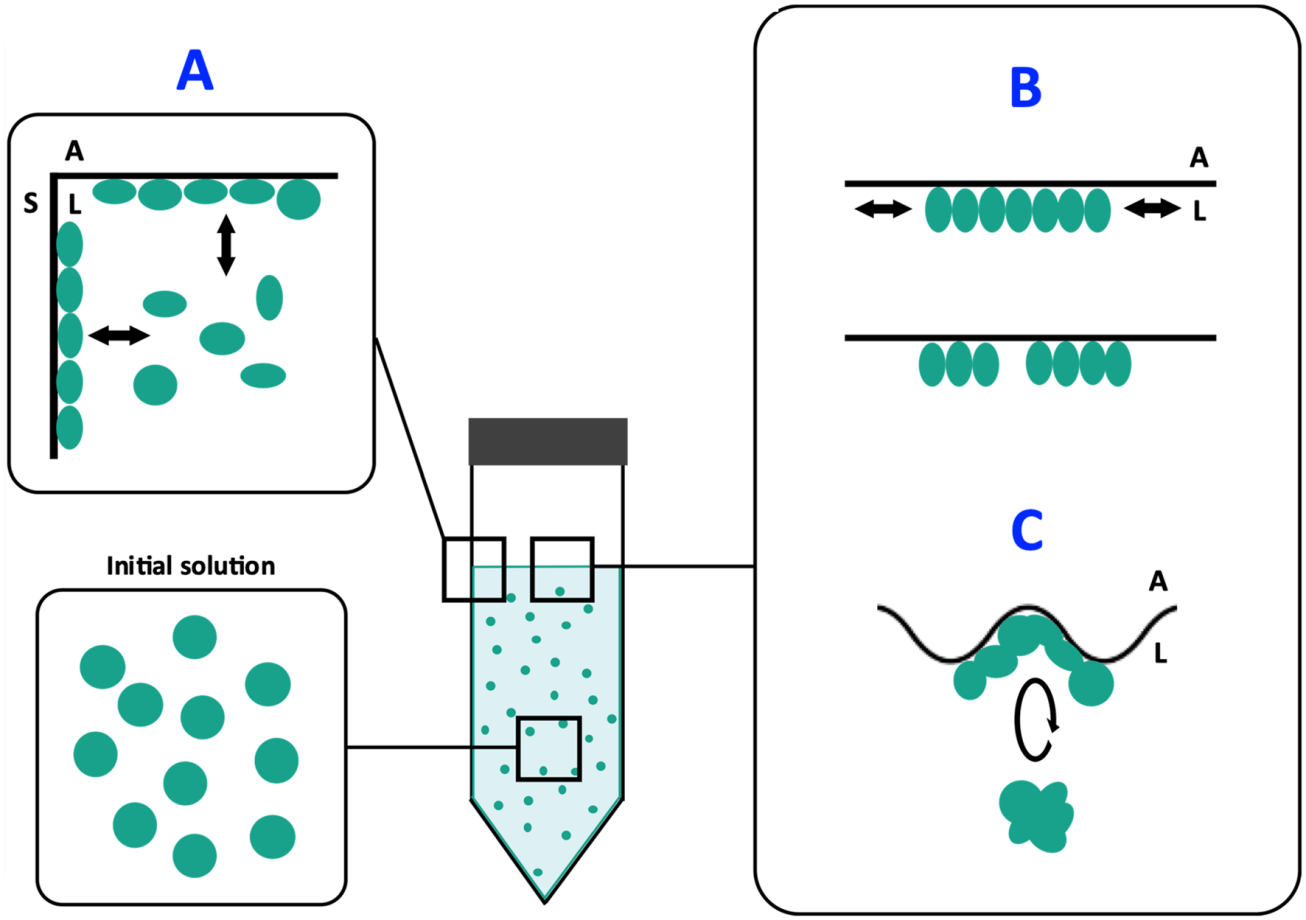
Model of protein destabilization by contact with plastic surfaces under agitation. (**A**) The affinity of the container surface for unfolded protein destabilizes the protein in solution through a conformational drift. This process is more important in case of fresh A/L and S/L interfaces. (**B**) The destabilized proteins adsorb at the A/L interface. Variations of interface quantity induce compression and decompression forces, promoting protein aggregation. (**C**) Shear forces may facilitate the transfer from and to interfaces in step A and break the protein films formed in step B. Adapted from Sluzky et al.^32^.

We propose a global model schemed in Fig. 10, with a concomitant action of adsorption on the plastic surface and other interfaces, adapted from Sluzky et al.^32^. In this scheme, strong binding materials and low binding materials would have the same effect by decreasing the cross talk between the material surface and the solution (Fig. 10 step A). It is indeed what was observed for TEFLON and LOBIND tubes with purified proteins (Fig. 1) and cell extracts (Fig. 2).

The fact that a significant α-syn loss is observed irrespectively of the material surface (but with a stronger effect with glass, Fig. 1) does not disagree with the mechanism presented in Fig. 10. Indeed, α-syn is partially unfolded in its native form so it cannot experience destabilization effect from the materials surface in Fig. 10A. Thus, α-syn is mainly destabilized by mechanical forces (Fig. 10, step B and C).

From this scheme, the limited BSA loss observed in Fig. 1 can be related to the very common use of BSA solutions for passivating plastic surfaces by adsorption. The passivating effect of BSA would indeed protect the rest of the solution from the material destabilization effect in Fig. 10A.

The specific vulnerability of BSA and α-syn in presence of glass (Fig. 1) may be due to their lower isoelectric point (see Table S1) that would promote electrostatic interactions with the material surface. In this case, we cannot exclude, in addition to surface destabilization, the presence on the material surface of aggregate nucleating sites^40^.

### Which proteins are sensitive to these combined stresses?

**Figure 11:**
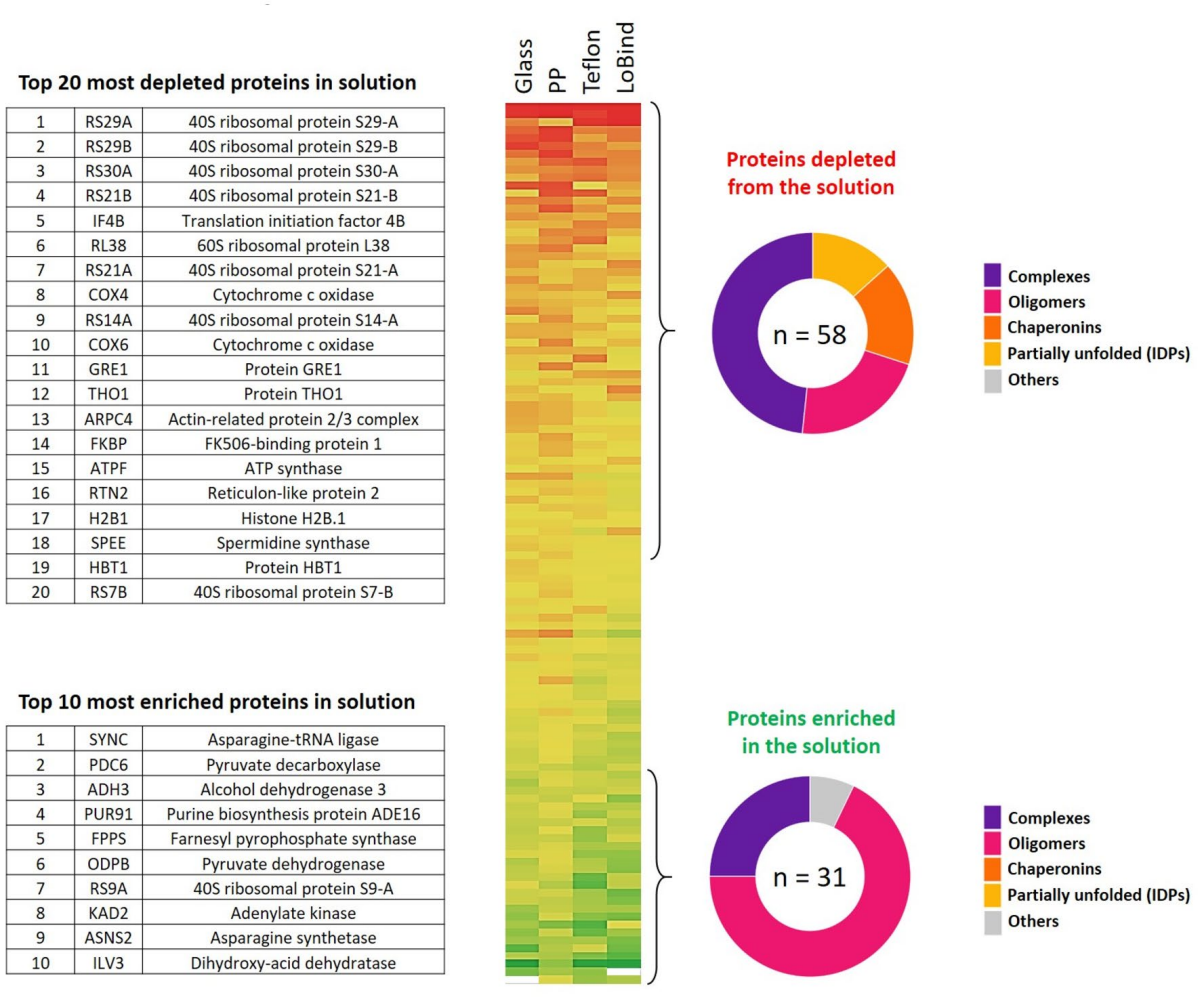
Quantitative proteomic analysis of protein depleted from and enriched in the solution after mixing YPE in PP, TEFLON, glass, LOBIND tubes at 3 rpm for 24 h at 6 °C. The color gradient indicates the status of the proteins: depleted (red) to enriched (green). The protein characteristics are issued from the Uniprot database^41^. A list of all considered proteins is given in supporting file Proteomic-SI2.

In order to identify the proteins which are the more sensitive to the imposed stress, we conducted a quantitative proteomic analysis before and after agitation of the YPE on the rotating wheel in PP, TEFLON, glass and LOBIND tubes, using the same conditions as shown in Fig. 2. The proteins that were depleted from the solution or enriched in the solution following agitation of YPE were identified by nanoLC-MS/MS (Fig. 11). Since the total protein concentration decreased after stress, we had to inject a larger volume of YPE solution in order to remain in the optimal sensitivity range for the analytical MS system. We applied a normalization coefficient corresponding to the loss of the samples calculated in molality scale in order to inject the same mass of proteins. The numbers given by the MS analysis are in molarity and a proper molarity to molality conversion would be, in principle, possible knowing the exact distribution in concentration of the analyzed proteins mixture. However, a significant proportion of the identified proteins have molarities between the LOD and LOQ (5 fmol). Thus, these proteins can be observed but not quantified and the molarity to molality conversion could not be applied (see supporting file proteomic-SI1). Therefore, as a safety margin, we decided not to consider proteins whose variations were of the order of the normalisation applied.

Using this method, we identified 112 proteins that exhibit a significant difference in concentration before and after agitation in one or several of the tested materials, based on Bayes statistical analysis (Bayes factor > 1) (see supporting file proteomic-SI2 for the protein list). Among these proteins, 58 proteins were highly depleted and 31 proteins were highly enriched, taking a threshold of minimum 15% concentration variation after mixing. No significant differences were observed as a function of the material, with most depleted and most enriched proteins observed for all the different types of surfaces. Highly enriched and highly depleted proteins were classified as complexes, oligomers, chaperonins, partially unfolded proteins or IDPs, and others (Fig. 11).

To validate the threshold of 15% to classify selectively enriched and depleted proteins in the MS data, a Monte-Carlo simulation test was performed (see M&M, Figure S9).

**Figure 12:**
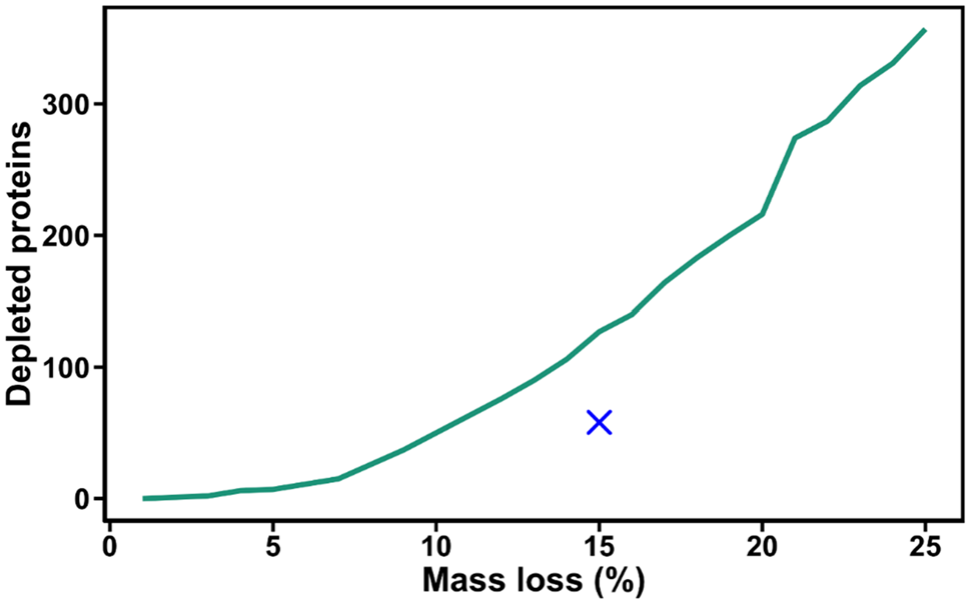
Calculated number of depleted proteins as a function of the mass loss. The result of the Monte Carlo simulations is represented by the green line. The measured protein loss and the number of depleted proteins identified by proteomic analysis for PP surface is shown by a blue cross.

During the simulations a mass loss target τ in the range of 0–25%, with a 1% increment, was applied non selectively to proteins. First, we computed the Bayesian factors (BF_sim_) for each protein of the full YPE using a target mass loss of τ % to state if the difference in quantity between the full extract and the depleted simulated sets was significant (BF_sim_ > 1) or not (BF_sim_ ≤ 1). The depletion of a given protein was assessed using a majority voting decision rule on 100 simulations. Proteins with simulated low abundances (quantities < 5 fmol) were removed in accordance to the LOD and LOQ determined experimentally. The results of the simulation for PP surface are shown in Fig. 12. The line depicts the simulated number of depleted proteins to achieved the target mass loss. For PP surface, to achieve a 15% mass loss, 125 proteins needed to be non selectively depleted, whereas only 58 are in the experiment. Our analysis shows also (supporting information part SF) that about 80% of the depleted proteins identified experimentally by proteomic show a stronger depletion than proteins from the simulated dataset. These two results demonstrate that protein depletions above 15% in the system are not due to a random process but rather to a selective depletion from the extract.

The depleted proteins fall in three main groups: partially unfolded proteins (or IDPs), chaperonins, and proteins forming large complexes (mainly ribosomes). The detection of proteins from the two first categories can be explained by the mechanism of protein adsorption at interfaces. Indeed, the adsorption process often implies a partial unfolding of the native protein that otherwise carries an enthalpy penalty associated with its secondary, tertiary and quaternary structure. By contrast, partially unfolded proteins such as IDPs do not suffer from this penalty and thus adsorb more easily^6,31^.

Chaperonins are also expected to absorb readily on interfaces, not directly but rather after interaction with adsorbed unfolded proteins for which they exhibit high specificity and affinity. Such mechanism has already been demonstrated for silica surfaces^31^.

The detection of large protein complexes (10–100 nm in diameter), such as ribonucleoproteins, is more surprising, since they are not usually overrepresented in adsorption experiments on inorganic surfaces^17^. On one hand, the partition coefficient between the A/L interface is not expected to be higher for large proteins compared to small ones^42^. Experimental data suggest that they could even be lower^43^. On the other hand, if proteins are considered as solid spheres, then their colloidal behaviour in solution depends on polydispersity, crowding and confinement. It was shown experimentally and by modelling that the diffusion of large proteins at interfaces is reduced compared to small ones. This mechanism could account for a longer time of residence and hence a higher number of protein complexes at the A/L interface, where they are subject to compression and elongation forces favouring further destabilization and aggregation^44^.

Recent cryoEM images of solutions of large proteins (25 nm in size, comparable to the size of the complexes identified here) demonstrated a rapid and quasi total adsorption at the A/L interface resulting in localized denaturation^45^ and complex dissociation^46^. This picture strongly suggests a distortion/elongation of the interfacial proteins induced by capillary forces (Scheme S4)^47^. This elasto-capillary effect can induce deformation in the proteins along the contact line whose order of magnitude, in the nm range, (see supporting information part SG) can be sufficient to break complexes apart, revealing hydrophobic surfaces that are prone to aggregation. Furthermore, capillary forces are expected to favour the pinning of larger particles at the A/L interface^48^, and their coalescence^49^.

A complementary explanation lies in the periodic compression-decompression induced by surface waves (Fig. 10)^27,50^ that add to the capillary constraints. Each time the A/L surface decreases, it induces an increase in the surface pressure of adsorbed proteins at this interface similar to the compression obtained in Langmuir trough experiments. Like capillary forces, the compression forces depend on the protein size and can reach 120 mN/m range (see supporting information part SG). This is strong enough to bring large proteins into contact and entangle them at the A/L interface^51^. Thus, these compression forces can probably favour the particle formation at the interface.

We must consider that these pressure fluctuations will be stronger and more frequent in wavy flow conditions, where the amount of surfaces varies rapidly and the energy dissipated in the protein structures (complexes, surface gels…) will then be much higher, due to the intrinsic viscosity of proteins^52,53^ and of protein assemblies^54^. Accordingly, wavy flow conditions will probably amplify the destructuring and entanglement processes occurring at the interfaces.

It is noteworthy that some proteins were significantly protected by the agitation effects, among which the small metabolic homo-oligomers (Fig. 11). Such assemblies are known to be more stable and less prone to aggregation^55^ even though it is difficult to understand how the shearing forces can stabilize them. One explanation may lie in the fact that such oligomeric complexes are kinetically stable^11^, *i.e.* they assemble faster than they disassemble. However, in diluted solutions, their assembly kinetics, which is controlled by the encounter of the components of the complexes, becomes the limiting factor for their stability. Any phenomena that can accelerate this encounter, such as shearing, may favour their stability.

Another explanation would be that these complexes are preferentially desorbed from surfaces by the shearing effect of turbulences. In some cases, shearing has been described as decreasing the amount of adsorbed protein, especially on hydrophobic surfaces^30,56^. The proteins that resist shear induced desorption form islands on the surface. This shows that lateral interactions between proteins on the surface are a key mechanism for adsorption under shear. Homo-oligomers, owing to their stability and lower exposed surfaces^57^, are less prone to such interactions and readily desorbed by the water flow. Therefore, it would be their limited adsorption on the surface under shear that would finally stabilize them upon exposure to combined stresses described here.

### How do these combined stresses compare to other destabilization processes?

Finally, we compared our results obtained with YPE cell extract with the data on the stability of purified BSA, Hb, and α-syn in light of the proposed mechanistic model (Figs. 1 and 10).

From the data presented in Table S1, most people would consider that Hb is more thermally and mechanically stable than BSA, and much more stable than α-syn. However, the Hb loss in the presence of PP, in the experiments presented in Fig. 1 is comparable with the one of α-syn and more important than the one of BSA. Therefore Hb, classified as a hard protein, seems comparatively more sensitive to the combined stress discussed here than to mild thermal or pure shear ones. Indeed, it takes three hours above the first melting temperature of Hb (50 °C) to achieve 5% losses^6,58^ whereas it can be achieved here with an energy (10 kJ) that, if it was used for heating, would only lead to 2 °C increase of the medium. On the contrary, BSA seems less sensitive to the combined stress than to a pure shearing, as it takes only 20 min of shear stress in the range 1000 s^−1^ to achieve a 4% loss^59^ that is only observed here after 24 h.

Even if less data are available on YPE stability, we must notice also that the ribosomal and RNA-binding proteins identified in the YPE as the more sensitive to the combined stress (supporting file proteomic-SI1 and SI2) are also the one identified as the more thermostable at a proteome scale^60^.

Our observations suggest therefore that the traditional stability scale of proteins, based mainly on thermal studies, cannot be applied to all types of stresses, and that, in specific conditions, proteins described as “soft and fragile” may be less sensitive and more long lived than “hard and stable” ones.

## Conclusion

In this work, we identified the disruptive effect of fluctuating air/liquid interfaces, potentiated by solid/liquid interfaces and shear stress under agitation in complex protein solutions. This effect is stronger for large protein complexes, yet small globular proteins are also affected. It is sufficiently strong to trigger the disruption of proteins like hemoglobin, leading to the formation of micron size gel-like protein aggregates in cellular extracts. As so many concurring effects are required to induce protein destabilization, it may seem surprising that protein destabilization occurs so often. However, such a combination is very common in protein handling. All protein solutions are stored or analysed in containers made of various materials during their production or their purification. Fluctuating interfaces can be observed when ripples form, that is as soon as these containers are moving. Furthermore, our observations and proposed mechanistic model also provide guidance on how to minimize these effects. Remove one of the components of the stress—material, air (even partially), or agitation—and proteins will be preserved.

## Material and methods

### Protein preparation and quantification

Yeast protein extracts were prepared from the *Saccharomyces cerevisiae* strain S288C (*Matα SUC2 mal mel gal2 CUP1*)^61^ with adaptations of the protocol previously described^15,16^. Cells were grown with shaking at 30 °C in a synthetic defined yeast medium (10 g L^−1^ of Bacto yeast extract, 20 g L^−1^ of Bacto peptone and 20 g L^−1^ glucose). Cells were collected by centrifugation, resuspended in phosphate buffered saline (PBS) containing 5% glycerol and a cocktail of protease inhibitors (1X EDTA-free from THERMOFISHER SCIENTIFIC and 1 mM PMSF) and broken using a French press. The cell extract was centrifuged (4000 rpm, 15 min, 4 °C and 14,000 rpm, 40 min, 4 °C) and the supernatant containing hydrosoluble proteins was recovered. The yeast protein extract concentration was determined using the 205 nm absorbance with an absorption coefficient of 31 L g^−1^ cm^−1^^62^.

Bovine Serum Albumin (BSA) was prepared using commercial lyophilized powder (SIGMA, A7030). It was dissolved in water to a concentration of 30 g L^−1^, dialyzed using a membrane with a 3500 kDa cut off, and centrifuged at 15,000 g for 5 min at 4 °C. The BSA concentration was determined by fluorescence spectroscopy (excitation: 279 nm, emission: 300 to 500 nm, slit width 4 nm, volume of the cuvette 200 µL). A calibration curve was realized using UV–visible spectroscopy (see Table S1) to convert the intensity of emission in concentration. For the calibration curve, 8 solutions containing from 12.5 to 100% of BSA diluted with milliQ water were realized. The sum of signal intensity from 330 to 340 nm has been calculated for each sample and plotted as a linear regression to obtain the proportionality constant between the signal intensity and the BSA concentration determined by UV–visible spectroscopy.

Porcine hemoglobin (Hb) was purified from pig blood supplied by the Harang slaughterhouse, Houdan, France, with agreement from the French Division Départementale de Protection des Populations. Fresh blood was immediately mixed with an anticoagulant solution composed of citric acid at 4.8 g L^−1^, sodium citrate at 13.2 g L^−1^ and dextrose at 14.7 g L^−1^ in Milli-Q water (1:4 volume of anticoagulant and blood). The blood was transported at 4 °C and purified immediately after arriving at the laboratory. The blood was first centrifuged (5000 *g*, 10 min, 4 °C) to remove the supernatant containing lipids by vacuum aspiration. A solution of NaCl at 9 g L^−1^ was used to resuspend the pellet with a glass rod to compensate for the removed volume while maintaining the ionic strength. The solution was centrifuged again (5000 *g*, 5 min, 4 °C) and the supernatant was removed. This step was repeated 3 times. The red blood cells were hemolyzed by osmotic shock by adding 1:3 volume of Milli-Q water at 4 °C. 2.8 M phosphate buffer pH 7.0 was added to precipitate the membranes at 4 °C. The solution was centrifuged (25,000 *g*, 20 min, 4 °C) and the supernatant containing free Hb was recovered. The supernatant was dialyzed 4 times for at least 2 h in 40 volumes of Milli-Q water at 4 °C through a membrane with a 3.5 kDa cut off. The dialyzed solution was centrifuged (25,000 *g*, 10 min, 4 °C). The solution was passed through an AG 501-X8 resin to remove the 2,3-bisphospho-glycerate bound to Hb and centrifuged (14,000 *g*, 15 min, 4 °C). The purified Hb solution was kept in filled and sealed tubes to avoid the presence of oxygen and the oxidation of Hb. Solutions were stored at 6 °C for one week and centrifuged (16,000 *g*, 5 min, 4 °C) before use. The absence of metHb and Hb concentration were measured by UV–vis spectroscopy (see Table S1).

For α-syn preparation, competent *E. coli* BL21(DE3) cells were transformed with the expression vector pRK172 encoding for human wild type α-syn, plated on LB agar Petri dishes containing 200 mg/L ampicillin and grown overnight at 37 °C. Transformed colonies were recovered in 6 × 1 L flasks of LB broth medium containing 200 mg/L ampicillin and grown at 37 °C under agitation (180 rpm). When cells reached an optical density of 0.6 at 600 nm, the expression of α-syn was induced by addition of 0.5 mM isopropyl-β-d-thiogalactopyranoside (IPTG) and further incubation at 37 °C and 180 rpm for 3 h.

Then, cells were harvested by centrifugation at 5000  *g* for 10 min at 4 °C, resuspended in 200 ml of 50 mM Tris–HCl pH 7.5 containing 1 mM PMSF and 2 cOmplete, EDTA-free protease inhibitor cocktail tablets (Roche) and frozen at − 80 °C.

For α-syn purification, cells were thawed in a water bath at 37 °C. After addition of 1 mM PMSF and 2 cOmplete tablets, cells were lysed by sonication on ice using a Sonics Vibra cell 750 Ultrasonic Processor (40% amplitude, cycles of 20 s sonication with 20 s pauses for a total sonication time of 300 s). Extracts were centrifuged at 40000  *g* for 25 min and the supernatant recovered. Ammonium sulfate powder (50% saturation i.e. 0.291 g/ml) was added at 4 °C under stirring to precipitate α-syn. Precipitated proteins were pelleted at 5000g for 25 min at 4 °C and resuspended in 400 ml of 50 mM TrisHCl pH 7.5 containing 2 complete tablets until complete pellet solubilization. 0.05% polyethyleneimine (Sigma) was added and the sample incubated for 30 min on ice to precipitate nucleic acids. The sample was then centrifuged at 5000  *g* for 25 min at 4 °C. The supernatant was loaded onto a XK 16/40 DEAE-Sepharose anion exchange column (GE Healthcare) (50 ml bed resin volume) equilibrated in 50 mM TrisHCl pH 7.5, 20 mM KCl, 1 mM β*-*Mercaptoethanol. After washing with 200 ml of the same buffer, elution was performed with a linear gradient of KCl (20 mM–1 M, 300 ml at a flow rate of 4 ml/min) and fractions of 4 ml were collected and stored at -80 °C.

Fractions of interest containing α-syn, identified by SDS-PAGE, were pooled and heated at 95 °C for 20 min to precipitate protein contaminants, while α-syn remained soluble.

The sample was centrifuged at 4000 *g* for 20 min at 4 °C and the supernatant recovered. The concentration of purified α-syn was determined spectrophotometrically (see table S1). Pure α-syn was filtered through sterile 0.22 µm nitrocellulose filters, aliquoted, flash-frozen in liquid nitrogen and stored at − 80 °C until use. Before experiments, α-syn was dialyzed against water and used in a 100 mM phosphate buffer (pH 7.0).

### Protein losses

All the experiments were realized in a phosphate buffer (100 mM, pH7) in a cold room (6 °C). For each experiment with YPE cell extract, three biological replicates and three technical replicates were measured for 27 measurements. Three replicates were measured for all the experiments with purified proteins. The percentage of protein loss are presented as the average and standard deviation.

The effect of the nature of the tube was studied at 0.1 g L^−1^ of proteins. The volume of liquid corresponds usually to 60% of the total volume of the tube. Four materials were tested: polypropylene (PP) (15 mL centrifuge tubes, FISHERBRAND), glass (PYREX), TEFLON (THERMOFISHER SCIENTIFIC), LOBIND (EPPENDORF). The surface area of each container was 50, 23, 85, 34 cm^2^ for PP, LOBIND, TEFLON, and glass tubes respectively. A cap of the same material was used except for the glass tubes for which the caps have a PTFE coating. The protein solutions were gently mixed on a rotating wheel with a 25 cm diameter, at fixed rotation speed for 24 h. Reference samples were prepared in the same conditions but were not submitted to agitation. The speed effect was studied with a 10 mL solution of YPE cell extract (0.1 g L^−1^) in PP tubes. A constant speed was set to 0, 3 and 30 rpm for 24 h. The protein concentration effect was studied with a 10 mL solution of YPE cell extract by mixing in PP tubes at 3 rpm for 4 h. The initial concentration ranged from 0.01 to 0.5 g L^−1^. The volume effect was studied with YPE cell extract at 0.1 g L^−1^ in PP tubes by mixing the solutions on a rotating wheel at 3 rpm during 24 h. Five volumes of solution were used: 1, 3, 10, 12.5 and 15 mL. The drying effect was studied by dip coating experiments. A PP porous membrane with a total surface area of 1 cm^2^ prepared from a disposable filter device (0.45 µm, Puradisc 25 PP, WHATMAN) was immersed and withdrawn from a 5 mL YPE solution at 0.1 g L^−1^ using a Dip Coater DC from KSV. The speed was set to 170 cm min^−1^ with a break of 20 s between each immersion and withdrawal (1 cm). The experiments were realized under magnetic stirring at room temperature during 24 h in a water-saturated chamber to limit evaporation. However, a slight loss of liquid was observed. Therefore, a correction was applied to the measured protein concentration. The reference samples were kept in the same environment without dip coating and magnetic stirring. Finally, the effect of an additional plastic surface was studied with a PP porous membrane (1 cm^2^) placed in 10 mL of YPE at 0.1 g L^−1^ and mixed during 24 h at 3 rpm on a rotating wheel.

### Protein desorption

The desorption experiments were realized using 10 mL YPE at 0.1 g L^−1^ in PP tubes. Three conditions were compared: (i) the solution was introduced and the desorption protocol was directly applied; (ii) the solution was stored at 6 °C during 24 h without mixing; (iii) the solution was mixed at 3 rpm during 24 h at 6 °C. The desorption protocol consists in successively immersing and filling the tubes in two beakers containing 2.5 L and 1 L of water respectively (120 s in each one). Using this method, protein drying on the surface was avoided. The total dilution factor was 4.10^6^. Then, 1 mL of a solution containing 0.1% v/v sodium dodecyl sulfate (SDS) was added to the tubes and mixed at 3 rpm during 1 h at room temperature. Because SDS slightly decreases the absorbance at 205 nm, the protein concentrations were corrected using a calibration curve measured with SDS. The efficiency of protein desorption at 0.1% of SDS was verified using the protein intrinsic fluorescence on plastic fibers (see Figure S4).

### Calculation of the amount of interfaces

The A/L/S triple interface length was measured directly on the tubes by picture analysis. For samples containing 1 and 15 mL of solution, the A/L and L/S interface surface area were determined by circular segment calculation. For other volumes, the meniscus at the A/L interface could be neglected and the A/L and L/S interface surface area were measured by picture analysis. The minimum and maximum values calculated correspond to different positions of the tube during mixing (horizontal or vertical).

### Dynamic light scattering (DLS)

DLS experiments were performed with a Zetasizer (MALVERN). After filtration with 1.2 µm filter, 400 µL were introduced in cells and, after 200 s of equilibrium at 25 °C, three measurements were done for three biological replicates at 25 °C. Correlograms were analyzed using a REPES algorithm supplied with the GENDIST software package^63,64^ in order to obtain an intensity distribution of characteristic times. The hydrodynamic radius (R_H_) was calculated according to the Stoke-Einstein law using a dynamic viscosity of 8.9 × 10^–4^ Pa s.

### Drop analysis

The contact angles and surface tensions of YPE on the different materials were measured with a Drop Shape Analyzer DSA 25 (KRÜSS) using Advance Surface software. The contact angles were measured with sessile drops. 5 µL were deposed on the substrate and the measurements were processed using an elliptical model after 60 s equilibrium. The surface tensions were measured with 20 µL pendant drops after 180 s equilibrium using the Young–Laplace model. The measured surface tensions of proteins are 48.0 ± 0.7 mN/m for YPE 25 g L^−1^ and 54.6 ± 1.3 mN/m for YPE 0.1 g L^−1^. The results correspond to the average and standard deviation of three replicates.

### Raman microscopy

The protein aggregates were imaged directly in solution by confocal Raman microscopy using a WITec alpha300 RA instrument (OXFORD INSTRUMENTS, Germany). 10 µL of solution were deposited between two fused silica windows (Esco Optics, USA) using a homemade Parafilm spacer in a closed Attofluor cell chamber (THERMOFISHER SCIENTIFIC, France). Raman images of 30 × 30 µm areas centered on the aggregates were acquired using 532 nm excitation wavelength, 100 × oil immersion objective (NA 1.3), 600 g/mm grating, 10 mW laser power, 0.2 s exposure time, and 0.3 µm step. The corresponding bright field image were taken in reflection mode with the same objective. Raman images of the protein aggregates were measured for two biological replicates. The analysis was repeated on at least 2 protein aggregates for each biological replicate. The Raman spectra of 0.1 and 25 g/L protein solutions were measured before and after mixing on a rotating wheel using a laser power of 10 mW, an exposure time of 5 s and 10 accumulations. A minimum of 5 spectra were measured and averaged for each sample. The absence of laser damage was controlled by accumulating single spectra on the same spot at the same power. Data were treated using WITec Project Five software. Cosmic rays were removed automatically and manually. The background was subtracted using polynomial fitting (rounded shape function of WITec software). Raman images were obtained by generating spectral components from the intensity distribution using automated component analysis and spectral demixing (True Component Analysis of WITec software). The average Raman spectrum of each component was extracted. Bright field and Raman images were exported to Fiji software.

### Proteomics

Tubes containing 60% of their maximum volume of YPE at 0.25 g L^−1^ in a phosphate buffer (100 mM) were mixed during 24 h at 3 rpm, 6 °C. The concentration was chosen to have optimal conditions for the detection and quantification. Proteomic experiments were performed at the Proteomic Analysis Platform of Paris Sud-Ouest (PAPPSO). YPE samples were deposited on SDS-PAGE gels and proteins separated using short migration time. A classic protein digestion protocol was applied (described in ref^65^). Samples were analyzed by LC–MS/MS on an Orbitrap Fusion Lumos Tibrid (THERMO SCIENTIFIC) mass spectrometer. The protein identification was performed using the *Saccharomyces cerevisiae* strain S288c protein database (^41^, 6750 entries, version 2020). The method followed was adapted from^66^ in order to obtain a semi absolute quantification. Quantification below 5 fmol were not considered as reliable. The variations of protein abundance were obtained by comparison with a reference sample exposed to the plastic, but not agitated. The mass spectrometry proteomics data have been deposited to the ProteomeXchange Consortium ^67^ via the PRIDE partner repository with the dataset identifier PXD038266.

To evaluate the specificity of the protein adsorbed on the polypropylene surface we developed the following Monte-Carlo simulation framework. Beforehand, we made these hypotheses: (1) if the mass depletion effect is non-specific, proteins in the cellular extract should be uniformly depleted; (2) proteins abundances (Q_*abs*_) are estimated using the semi-quantitative method described in^66^; (3) protein abundance variability used for simulations are estimated using the experimental deviations of the four technical replicates. The overall simulation strategy is depicted in Figure S9.

The goal is to generate four replicates, starting from the four samples of full YPE shotgun quantification, that were depleted to a target mass loss of τ %. For each protein detected by LC–MS/MS: (1) we calculated the semi quantitative index *Q*_*abs*_ for each of the four replicates; (2) these concentration measurements were converted into molecule sets of size *N* = *Q*_*abs*_* x Avo*_*coef*_ where *Avo*_*coef*_ = 5.10^6^ is an Avogadro like number; (3) these molecule sets (one per protein) were randomly sampled and the size of the set decreased by one unit. This sampling process was repeated until an overall mass loss of τ% is obtained. (4) Then, down sampled molecule sets were converted back to the semi quantitative index *Q*_*abs*_. (5) For each protein, the *Q*_*abs*_ values of the four full YPE replicates and the four simulated molecule set replicates were compared using a Bayesian version of the *t*-test^68^. The overall process, from step 2 to 5, was repeated 100 times.

### SAXS

The specific surface area of the filter was measured by Small Angle X-ray Scattering (SAXS). The presented data correspond to an average of 2 measurements under vacuum, carried out on a Xeuss 2.0 cupper setup from XENOCS with 3600 s counting times and a sample-to-detector distance of 2.5 m.

## Supplementary Information

Supplementary Information 1.

Supplementary Information 2.

Supplementary Information 3.

## Data Availability

The mass spectrometry proteomics data have been deposited to the ProteomeXchange Consortium via the PRIDE partner repository with the dataset identifier PXD038266. The full protein lists that exhibit a significant difference in concentration before and after agitation are also available in SI (proteomic-SI1 and SI2). The other data is available from corresponding authors on request.
