# Supplementary material for: A proteome scale study reveals how plastic surfaces and agitation promote protein aggregation
Source: Sci Rep. 2023 Jan 21;13:1227. doi: 10.1038/s41598-023-28412-7 (PMC9867740; doi:10.1038/s41598-023-28412-7)
Supplement: Supplementary file 3 — Supplementary Information 3. [file 41598_2023_28412_MOESM3_ESM.docx]

**Supporting Information**

**A proteome scale study reveals how plastic surfaces and agitation promote protein aggregation**

Marion Schvartz^1,2^*, Florent Saudrais^1^, Stéphanie Devineau^3^, Jean-Christophe Aude^4^, Stéphane Chédin^1,4^, Céline Henry^5^, Aarón Millán-Oropeza^5^, Thomas Perrault^2^, Laura Pieri^4^, Serge Pin^1^, Yves Boulard^4^, Guillaume Brotons^2^, Jean-Philippe Renault^1^*.

* Corresponding authors

^1^ Université Paris-Saclay, CEA, CNRS, NIMBE, LIONS, 91191 Gif-sur-Yvette, France

^2^ Institut des Molécules et Matériaux du Mans (IMMM), UMR 6283 CNRS, Le Mans Université, Avenue Olivier Messiaen, 72085 Le Mans Cedex, France

^3^ Université Paris Cité, CNRS, Unité de Biologie Fonctionnelle et Adaptative, F-75013 Paris, France

^4^ Université Paris-Saclay, CEA, CNRS, Institute for Integrative Biology of the Cell (I2BC), 91198, Gif-sur-Yvette, France

^5^ Université Paris-Saclay, INRAE, AgroParisTech, Micalis Institute, PAPPSO, 78350, Jouy-en-Josas, France

**Table of contents**

(SA) Properties of protein used

(SB) Complementary material information

(SC) Protein loss without agitation, using deep coating or extra surfaces and associated schemes

(SD) Hydrodynamic considerations

(SE) Complementary Raman data

(SF) Complementary Proteomic information

(SG) Evaluation of the elastocapillary and compression forces and associated scheme

**(SA) Properties of protein used**

| protein | species | MW (kDa) | pI | PDB | SCOP class | Adsorption coefficient (wavelength) | Denaturation Enthalpy (kJ/mol) | Damaging shear stress (s^-1^) |
| --- | --- | --- | --- | --- | --- | --- | --- | --- |
| Hb | pig | 64.78 | 7.0^(1)^ | 1qpw | all alpha | 60.6 10^3^ L.mol^-1^.cm^-1^  (576 nm) ^2^ | 120 ^(3)^ | >>10000 ^(4)^ |
| BSA | bovin | 66.64 | 4.7^(5)^ | 4f5s | all alpha | 44 10^3^ L.mol^-1^.cm^-1^  (280 nm) ^6^ | 90 ^(7)^ | 1000-10,000 ^(8)^ |
| Alpha Syn | human | 14.48 | 4.7^(9)^ | 1xq8 | coiled coil | 5.96 10^3^ L.mol^-1^.cm^-1^  (280 nm)^*^ | n.d.^**^ | <100 ^(10)^ |

Table S1. Main properties of Hemoglobin, Serum albumin and alpha synuclein. *α-syn absorption coefficient was calculated using ProtParam ^(11)^ **α-syn being mainly disordered, has no denaturation enthalpy. ^(12)^


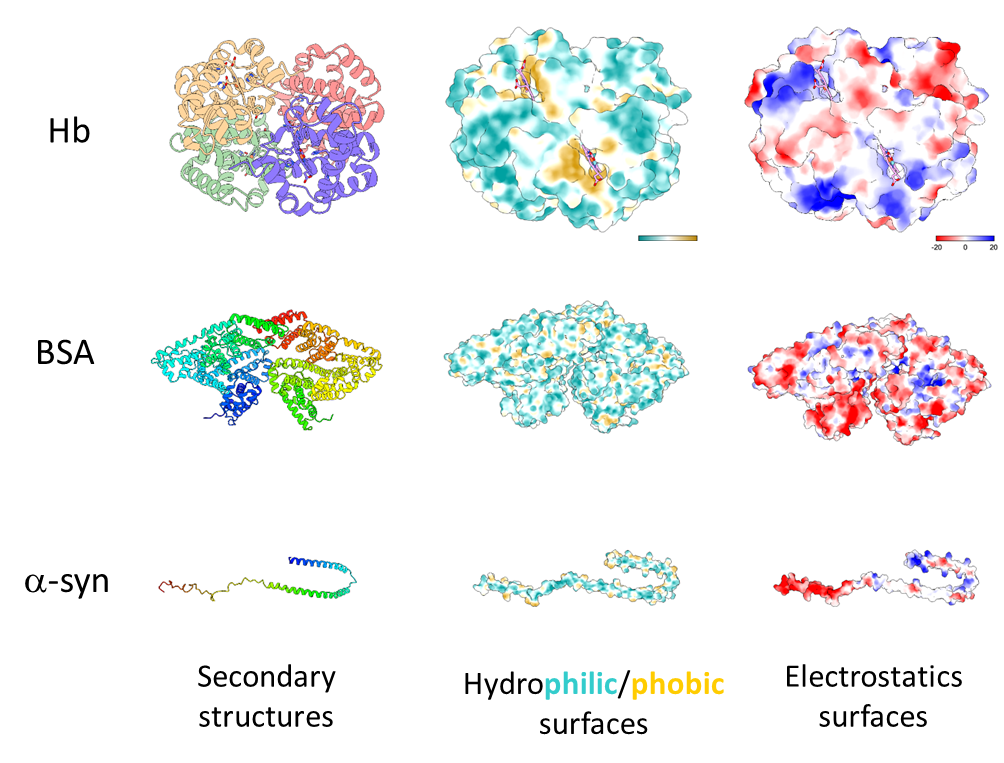


Table S2. Structural comparisons of Hemoglobin, Serum albumin and alpha synuclein, based on their pdb structures. Figures prepared using ChimeraX ^13^

**(SB) Complementary material informations**


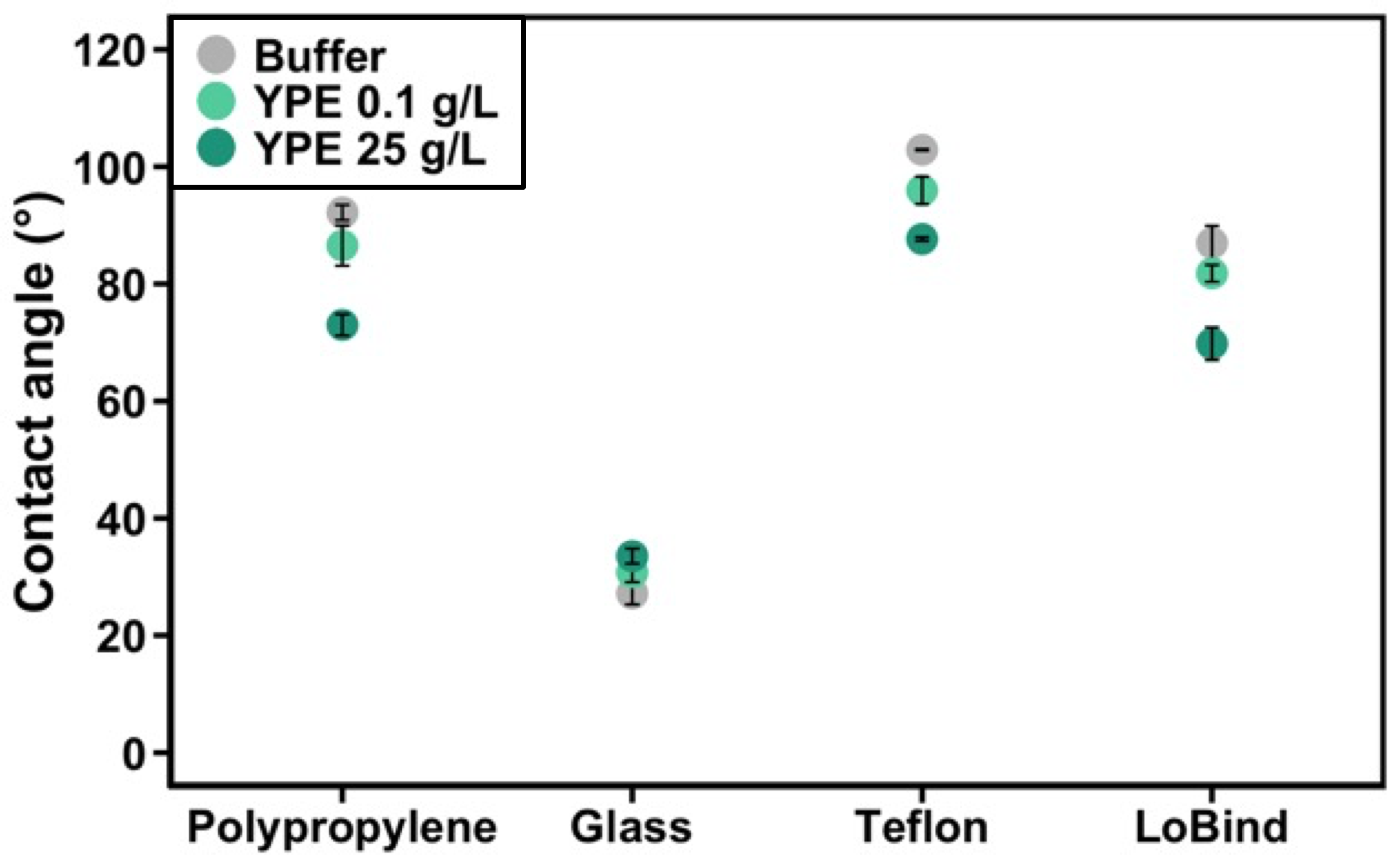


Figure S1. Contact angle of YPE solutions at 0.1 g/L (light green), 25 g/L (dark green) and of phosphate buffer (grey) on four substrates: PP, glass, Teflon, LoBind ^TM^.


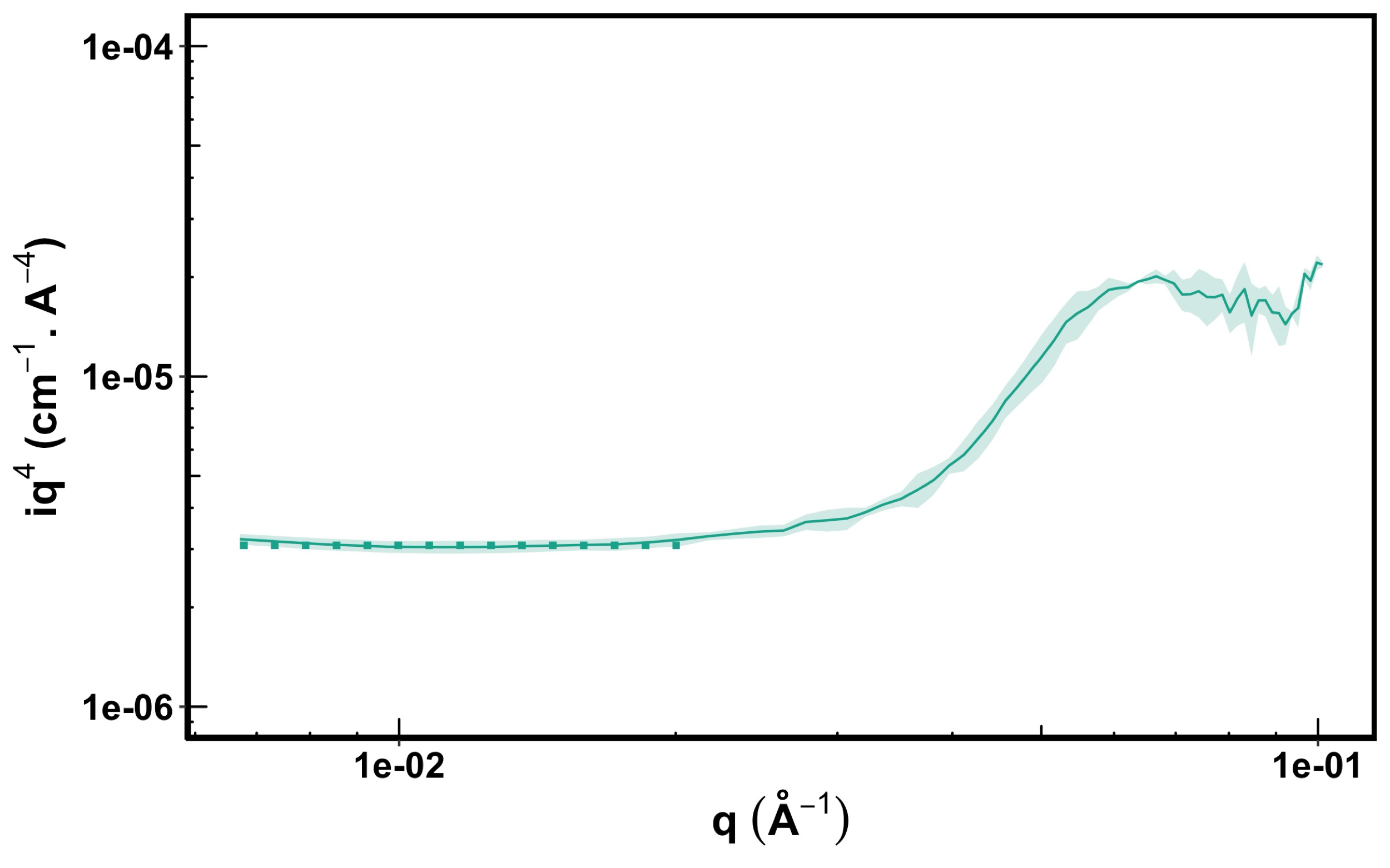


Figure S2. SAXS measurement of PP filter under vacuum, carried out on a Xeuss 2.0 cupper setup from Xenocs ^TM^ with 3600 s counting times and a sample to detector distance of 2.5 m. Corresponds to an average on two measurements.

**(SC) Protein loss without agitation, using deep coating or extra surfaces and associated experimental schemes**

|  | Polypropylene | | Glass | | | Teflon | | | LoBind | | |  |
| --- | --- | --- | --- | --- | --- | --- | --- | --- | --- | --- | --- | --- |
|  | 0 rpm | 3 rpm | | 0 rpm | 3 rpm | | 0 rpm | 3 rpm | | 0 rpm | 3 rpm | |
| BSA-AlphaSyn | 6.4E-04 | 2.6E-04 | | 6.7E-04 | 5.0E-01 | | 8.1E-01 | 4.8E-05 | | 1.6E-02 | 5.3E-06 | |
| Hb-AlphaSyn | 5.4E-01 | 2.9E-03 | | 8.9E-01 | 6.9E-03 | | 2.6E-01 | 4.3E-05 | | 1.0E+00 | 2.9E-06 | |
| Hb-BSA | 3.0E-04 | 3.9E-02 | | 4.8E-04 | 2.5E-02 | | 5.3E-01 | 9.8E-01 | | 1.6E-02 | 2.3E-01 | |

Table S3. Statistical analysis of the results associated to the figure 1. These p-value were determined using the Tukey HSD test realized from variance analysis (Anova).

| Material | Protein loss without agitation (%) |
| --- | --- |
| PP | 0-0.5 |
| Glass | 0-0.5 |
| Teflon | 0-2 |
| LoBind | 0-0.5 |

Table S4. Measurement of protein loss after mixing of YPE in PP, glass, Teflon and LoBind tubes without agitation after 24h.

|  | Protein loss (%) |
| --- | --- |
| Without dip coating | 0-4 |
| With dip coating | 0-4 |

Table S5. Measurement of the protein loss from YPE on PP filter in the dip coating experiment, with and without motion.

|  | Without PP filter | With PP filter |
| --- | --- | --- |
| Protein loss (%) | 19 ± 1 | 41 ± 3 |

Table S6. Measurement of protein loss from YPE in PP tubes with and without PP filters.


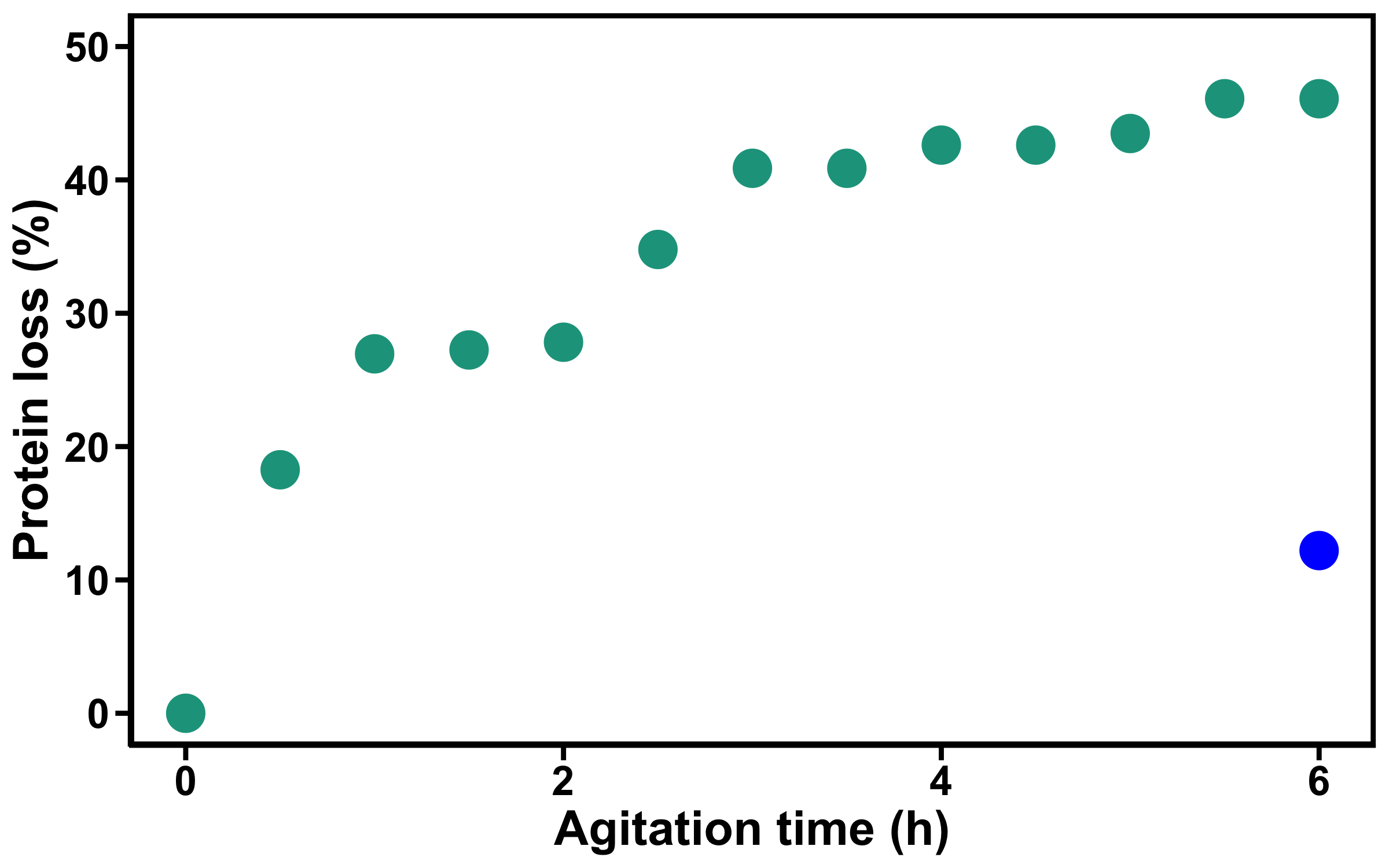


Figure S3. Protein loss (%) according to the agitation time (3 rpm, 6 °C) of a YPE solution at 0.1 g.L^-1^ in a phosphate buffer (pH7, 100 mM) in a PP tube containing 10 mL. The tube is changed to renew A/L and S/L interfaces each 30 min. The concentration is measured at each tube change. The blue point corresponds to a sample mixed during 6 h without tube change.


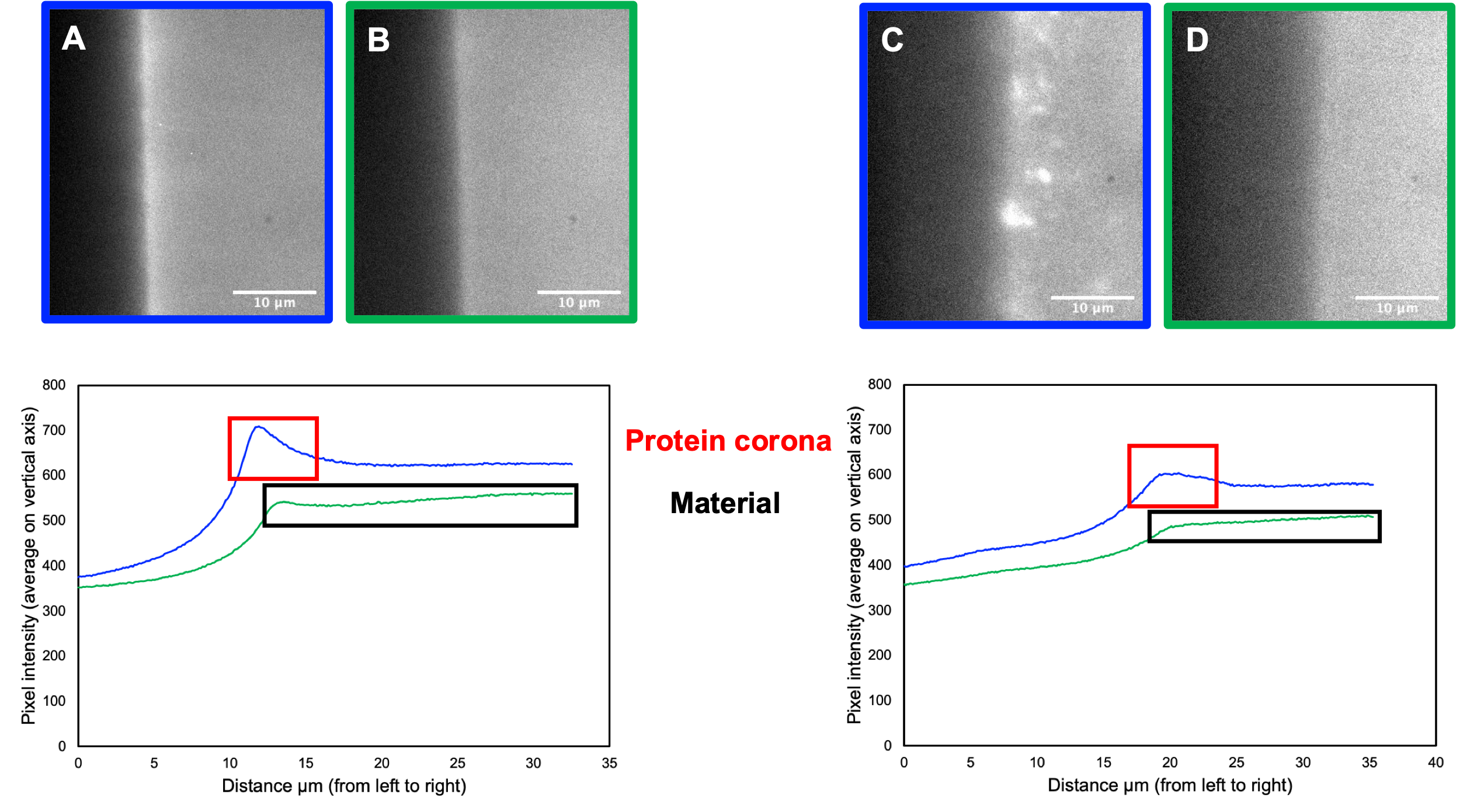


Figure S4. Fluorescence imaging (DISCO beamline, synchrotron SOLEIL) of yeast protein extract (23g/L) on plastic fibers (excitation: 265 nm, emission: [329-351 nm]) without adsorbed aggregates (A), or with adsorbed aggregates (B) before (A, C, blue line) and after (B, D, green line) washing with SDS 0.1% during 30 min.


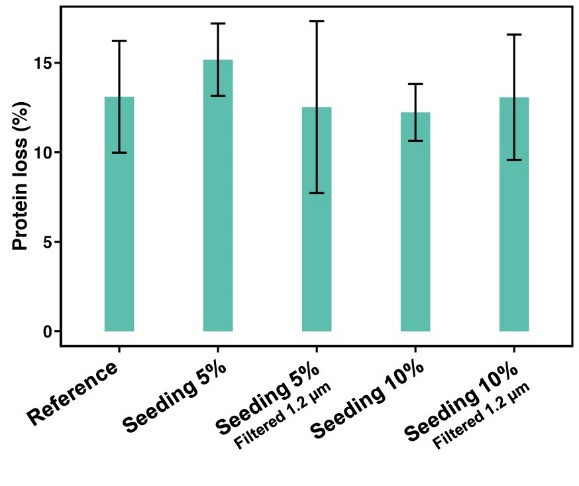


Figure S5. Protein losses after seeding at 5 and 10% of an aged solution filtered (1.2 µm) or not (YPE 0.1 g.L^-1^, phosphate buffer 100 mM, pH7, mixed at 3 rpm during 24 h at 6°C in polypropylene tubes filled at 60%) in fresh cell extracts. The protein concentrations are measured before and after mixing in the same conditions than the aged solution. The error bars correspond to the standard deviation for three biological replicates.

Schemes of the experimental set-up used in protein loss studies (S1, S2, S3). (S1) Rotating wheel experiment. The A/L interface is represented as a function of the volume of liquid and the tube position. (S2) Dip coating experiment. (S3) Experiment with PP filter inside a PP tube.


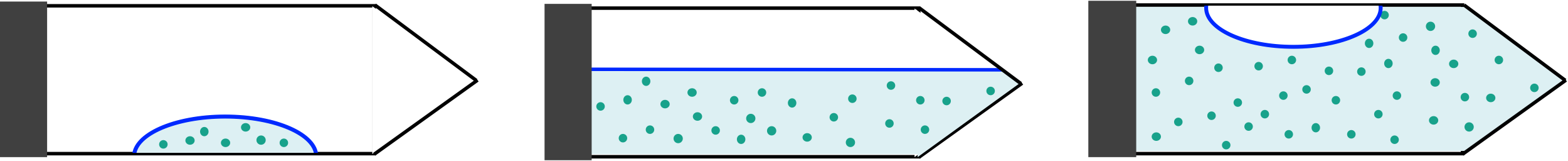


Scheme S1


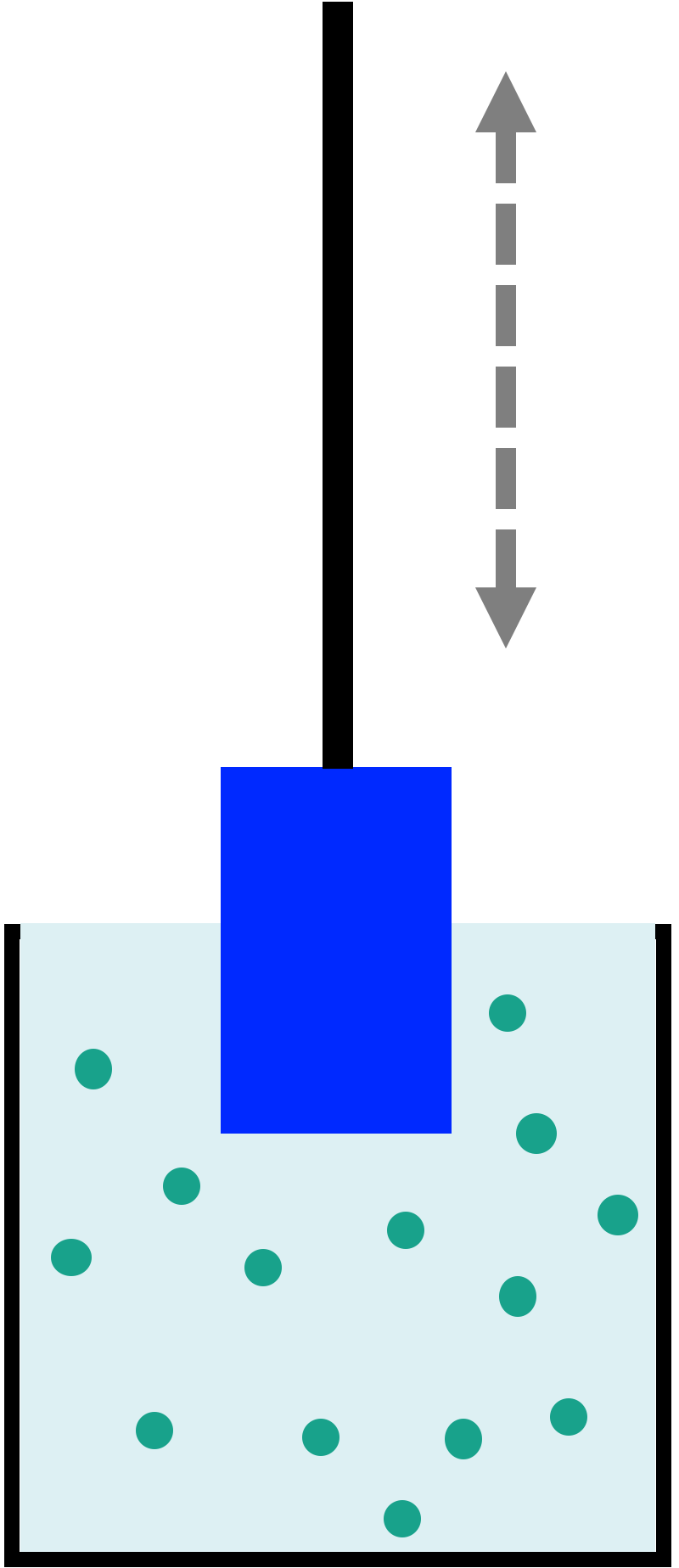


Scheme S2


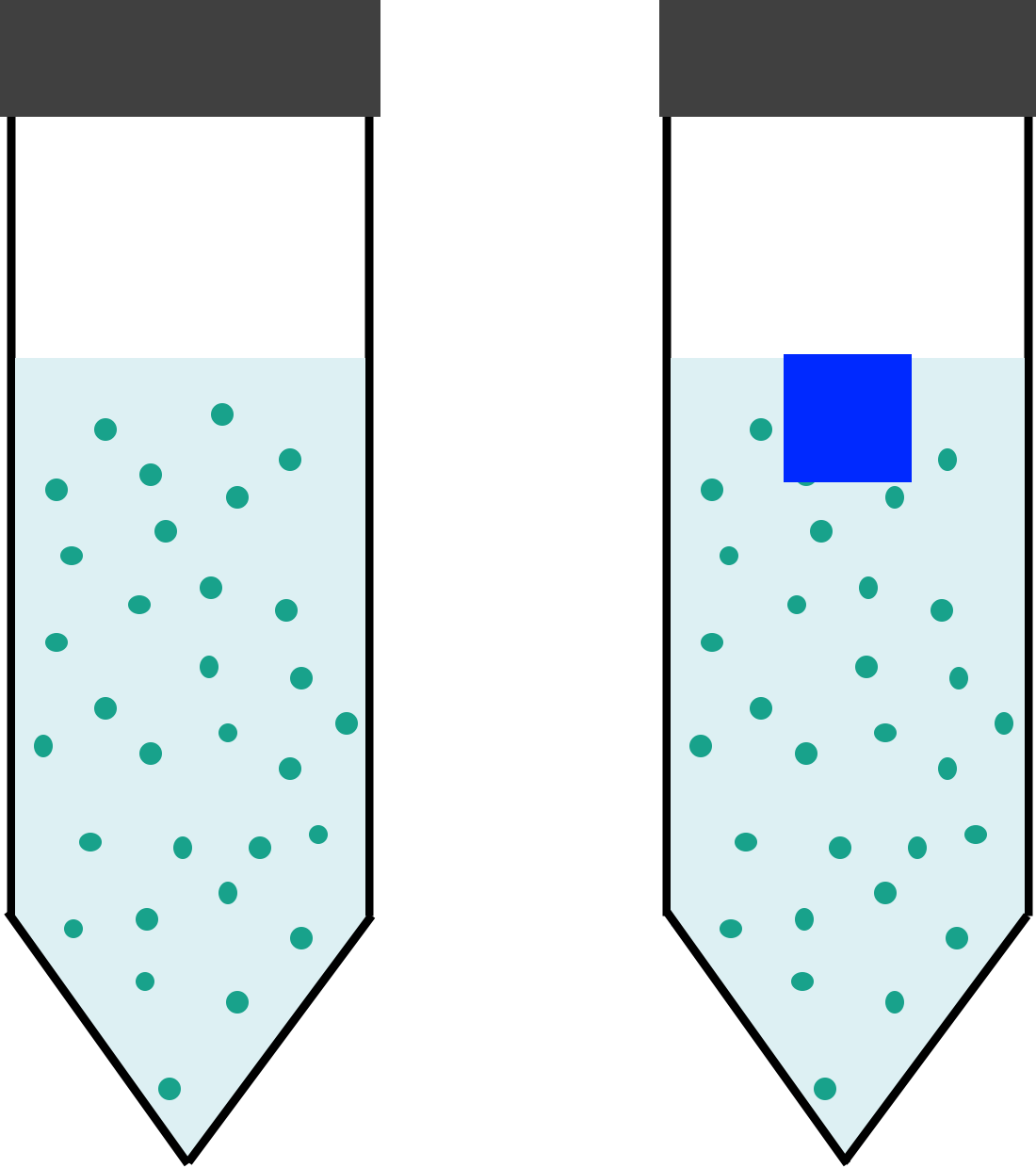


Scheme S3

**(SD) Hydrodynamic considerations**

| **Parameters** | **Equation** | **Numerical value** |
| --- | --- | --- |
| Kolmogorov length scale η (m) | ≅ (υ^3^/ε)^0.25^ | 30 µm (ε = 0.5 W/kg) |
| Kolmogorov strain rate (s^-1^) | ≅ (ε/υ)^0.5^ | 700 s^-1^ (ε = 0.5 W/kg) |
| Expected shear force on a particle of radius R | ≅ ρ (ε.υ)^0.5^R^2^ | 4x10^-16^ N (R = 25 nm)  4x10^-18^ N (R = 2.5 nm) |

Table S7. Kolmogorov length scale and strain rate associated to the turbulence occurring in the experiments. ε is the average rate of dissipation of turbulence kinetic energy per mass unit mass (m^2^ s^-3^), υ the kinematic viscosity of the fluid (υ = 1x10^-6^ m^2^ s^-1^ for water at 20°C), ρ the fluid density (ρ = 1000 kg m^-3^).


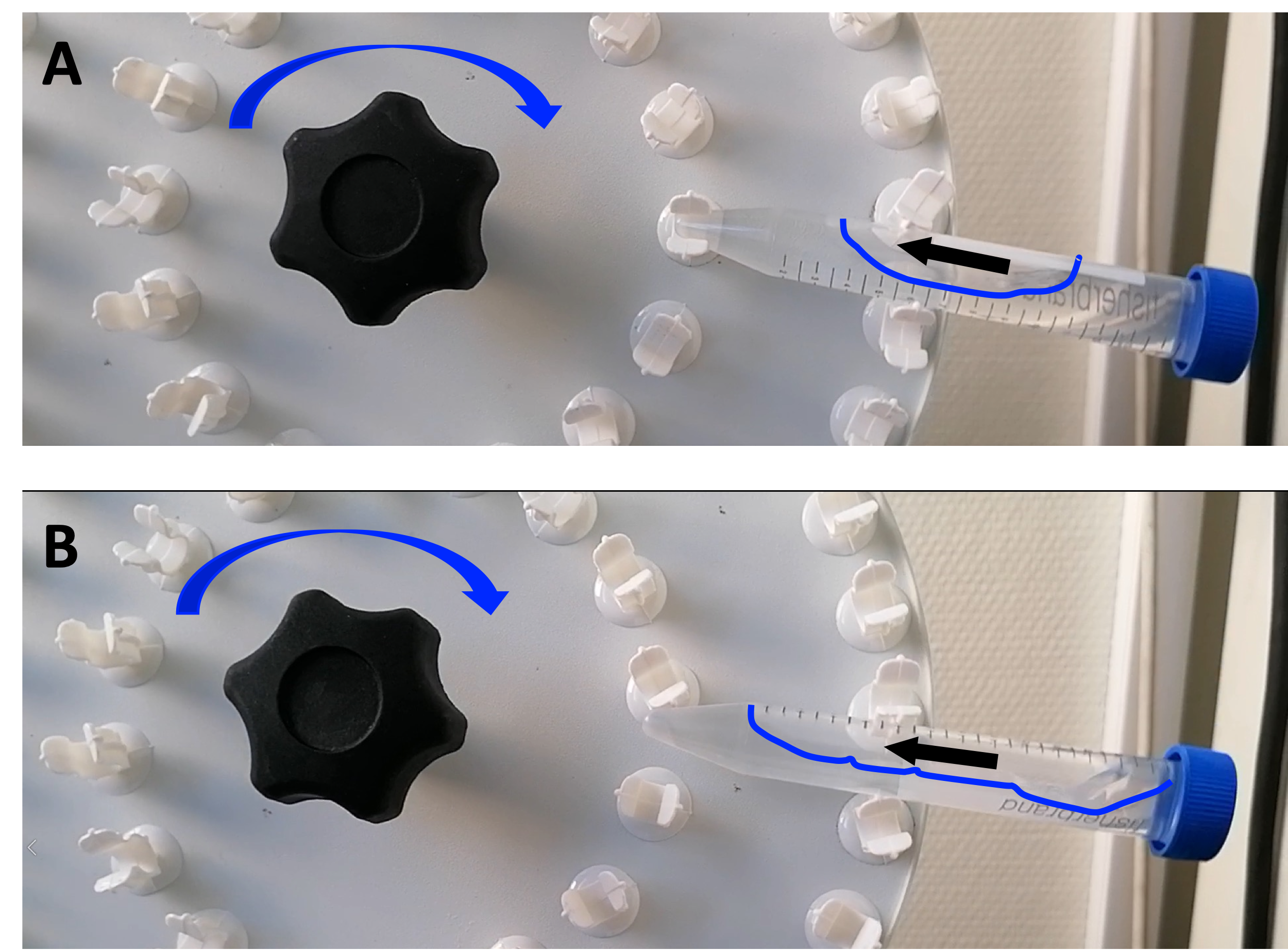


Figure S6. Pictures of the solutions in PP tubes during mixing on the rotating wheel to analyze the flow patterns. The A/L interface is highlighted in blue. (A) Plug flow: only one ripple is visible in the moving bubble. (B) Wavy stratified flow: multiple ripples are observed and the rear of the bubble is markedly more turbulent.


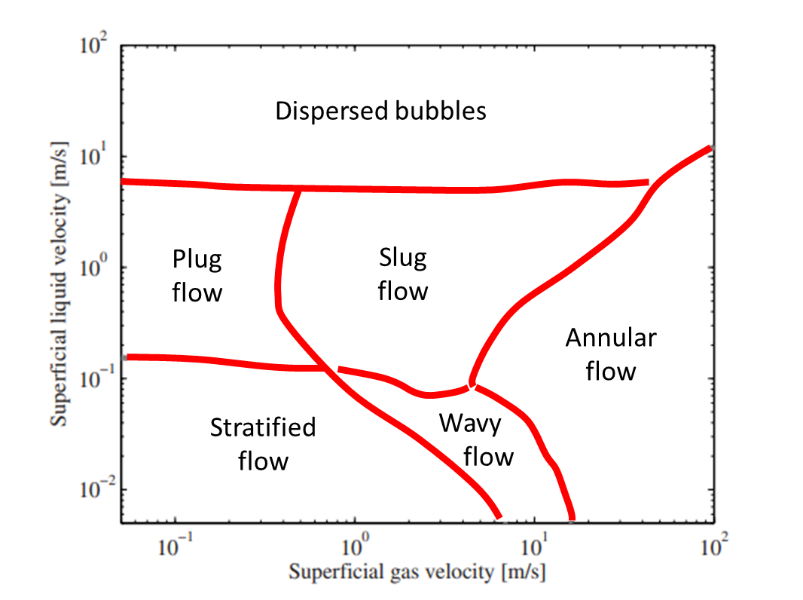


Figure S7. Domains of plug flow and slug flow as a function of the gas and liquid velocity, adapted from ^14^.

**(SE) Complementary Raman data**


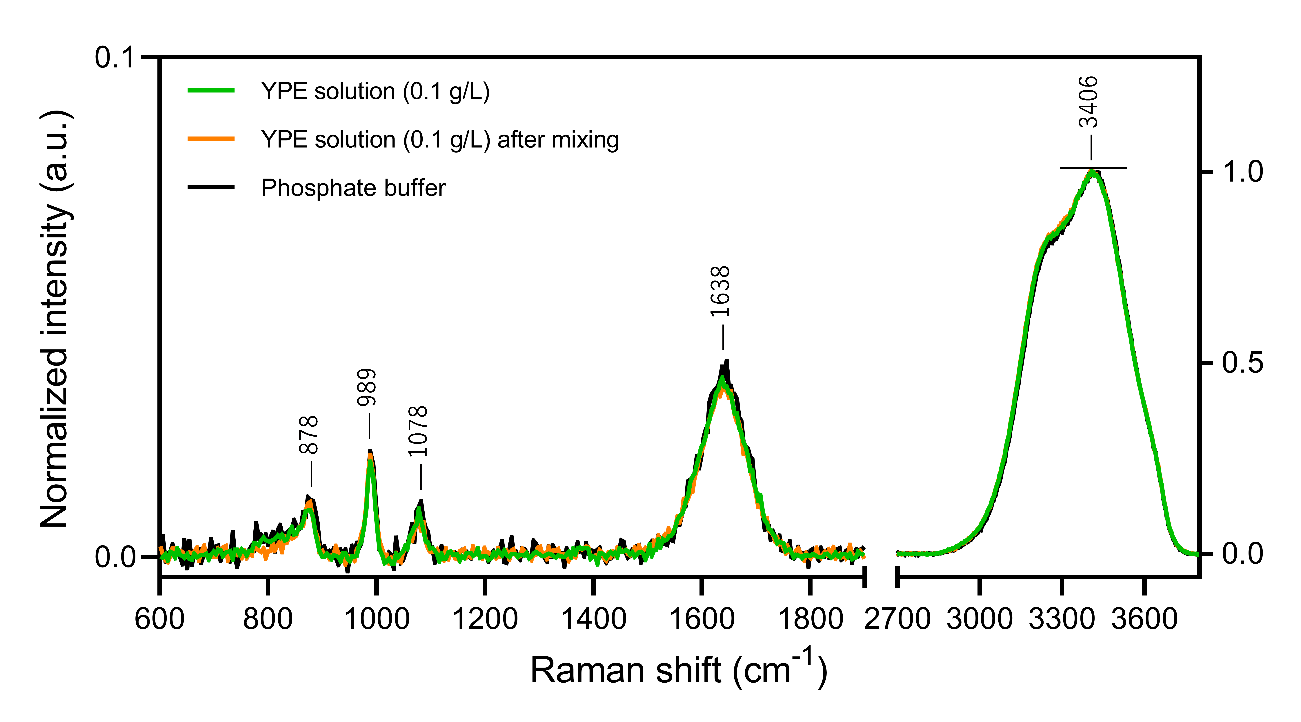


Figure S8. Raman spectra of 100 mM phosphate buffer pH 7 (black) and YPE solutions at 0.1 g/L in phosphate buffer before (green) and after (yellow) mixing. The spectra were normalized to the OH band of water at 3406 cm^-1^.

| Band (cm-1) | Attribution | Référence |
| --- | --- | --- |
| 1665 | Amide I | ^15^ |
| 1002 | Phenylalanine aromatic ring,  C-C stretching |  |
| 1603 | Phenylalanine aromatic ring,  C=C bending |  |
| 1170 | Tyrosine C-H bending. |  |
| 883 | CH_2_ rocking of proteins |  |
| 1450 | CH_2_ bending of proteins |  |
| 2935 | CH_2_ asymmetric stretching of proteins and lipids |  |
| 1124 | CH_3_ rocking and CCH bending at 1124 cm^-1^ |  |
| 726 | Adenine breathing mode of DNA or RNA | ^16^ |

Table S8. Attribution of bands present in the aggregates (figure 8A)

| Band (cm-1) | Attribution | Reference |
| --- | --- | --- |
| 678 | Heme ν_7_ (resonant)  possibly flavohemoprotein YHB1 identified in proteomic | ^17^ |
| 713 | porphyrin in cytochrome complexes | ^18^ |
| 853 | polysaccharides or phospholipids | ^19^ |
| 926, 1058 | Not determined |  |
| 1417 | C=C stretching of quinoid rings, possibly quinones. | ^15^ |

Table S9. Attribution of bands missing in the aggregates (figure 8B)

**(SF) Complementary Proteomic information**

The mass spectrometry proteomics data have been deposited to the ProteomeXchange Consortium (<http://proteomecentral.proteomexchange.org>) via the PRIDE partner repository with the dataset identifier PXD038266.


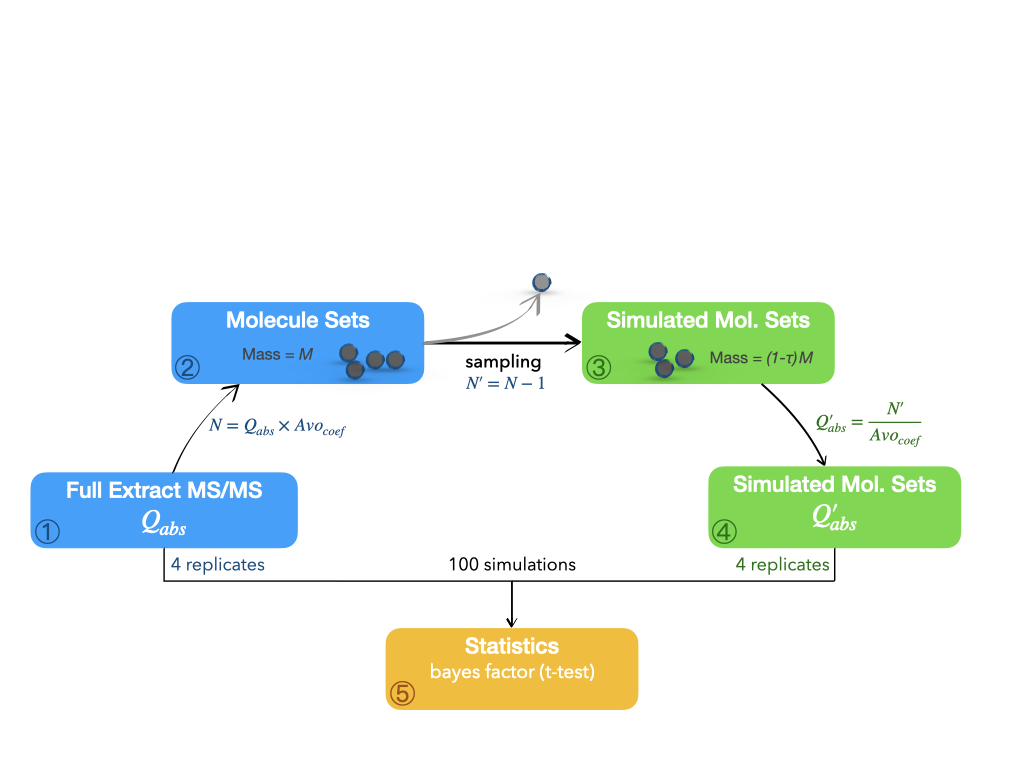


Figure S9. Simulation strategy used to evaluate the specificity of protein adsorbed on PP surface by Monte-Carlo simulation.

We determined whether any type of protein could contribute to the mass loss, or if some proteins show a higher affinity compared to others. Indeed, some of the depleted proteins could also be depleted in the simulations (that is randomly depleted). To determine if the strength of the depletion is the same in both cases, we developed an alternative approach using Z-scores. In this method, for a protein $i$*,* we compared the Bayesian factor of the *observed* level of depletion on PP surface ($BF_{\mathrm{pp}}^{i}$) to the one of the simulations ($BF_{sim}^{i}$) using the Z-score formula (eq. 1):

$Z^{i}=\frac{BF_{pp}^{i}-\overline{BF_{sim}^{i}}}{\sigma\left( BF_{sim}^{i} \right)}$ (eq. 1)

Where $\overline{BF_{sim}^{i}}$ and $\sigma\left( BF_{sim}^{i} \right)$ are the average and the standard deviation of the Bayesian factors for protein $i$ in 100 simulations respectively. We used the classical threshold $Z^{i}>3$ to consider that the depletion strength for protein $i$ is stronger in experiment compared to the simulation. This analysis shows that 79 % of the depleted proteins identified experimentally by proteomic show a stronger depletion than proteins from the simulated dataset for PP surface.

**(SG) Evaluation of the elastocapillary and compression constraints and associated schemes**


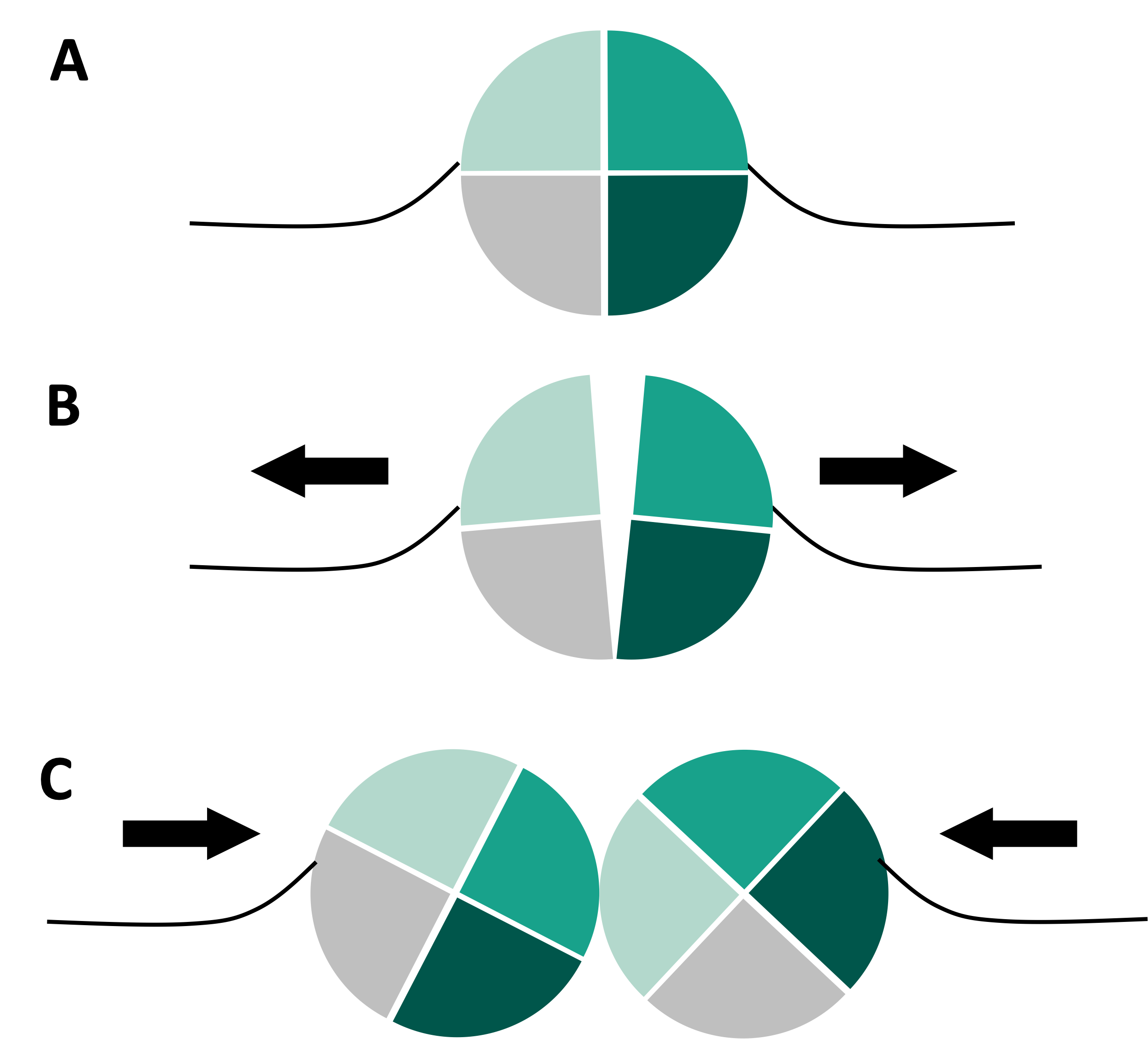


Scheme (S4). (A) Initial state (B) Elastocapillary forces tend to break larges complexes appart. (C) Decrease on surface tends to bring proteins together, bypassing the electrostatic repulsion.

In case B, the order of magnitude of the constraint Δ on the protein along the contact line can be evaluated using equations S1 and S2: ^20^

Δ_horizontal_ = γ cos(Θ) (eq. S1)

Δ_vertical_ = γ sin(Θ) (eq. S2)

Where γ is the solution surface tension, θ is the contact angle between water and protein solution. θ depends on the surface residues and thus is heterogeneous depending on the protein surface. ^21^ Owing to the fact that the norm of the constraint is independent of the contact angle, and thus of the surface tension γ_prot_ (here γ_prot_ = 55 mN/m for the protein solution used, see M&M), it can be used as a proxy for the constraint Δ. For monomeric proteins, the Young modulus is in the GPa range, and the expected deformation are on the sub Å level. However, for complexes, the Young modulus is ten times lower due to weaknesses at the monomer-monomer interfaces. ^22^ Therefore the characteristic size, and accordingly the involved forces, are larger for protein complexes. In this condition, the deformation at the A/L interface can reach a nanometer, *i.e.* the interaction between components of the complexes are broken,

In case C, the surface pressure P at rest can be evaluated through the surfactant effect of proteins, following Ramos et al. ^23^ (eq. S3):

P = γ_wat_ -γ_prot_ (eq. S3)

Where γ_wat_ is the surface tension of water (γ_wat_ = 72 mN/m as measured here). The pressure increase is inversely proportional to the surface decrease during a compression step, here reaching 120 mN/m (see variations of A/L in Table 1). Like capillary forces, the compression forces depend on the protein size and are in the nN range for a 10 nm protein. These forces are much larger than the one measured on different systems in the 10-100 pN range ^24^ ^25,26^ and known to induce protein and complexes collapses.

1 Hempe, J. M. & Craver, R. D. Separation of hemoglobin variants with similar charge by capillary isoelectric focusing: Value of isoelectric point for identification of common and uncommon hemoglobin variants. *ELECTROPHORESIS* **21**, 743-748, doi:<https://doi.org/10.1002/(SICI)1522-2683(20000301)21:4><743::AID-ELPS743>3.0.CO;2-1 (2000).

2 Banerjee, R., Alpert, Y., Leterrier, F. & Williams, R. J. P. Visible absorption and electron spin resonance spectra of the isolated chains of human hemoglobin. Discussion of chain-mediated heme-heme interaction. *Biochemistry* **8**, 2862-2867, doi:10.1021/bi00835a025 (1969).

3 Sanchez-Guzman, D. *et al.* In Situ Analysis of Weakly Bound Proteins Reveals Molecular Basis of Soft Corona Formation. *ACS Nano* **14**, 9073-9088, doi:10.1021/acsnano.0c04165 (2020).

4 Duerkop, M., Berger, E., Dürauer, A. & Jungbauer, A. Impact of Cavitation, High Shear Stress and Air/Liquid Interfaces on Protein Aggregation. *Biotechnology Journal* **13**, 1800062, doi:<https://doi.org/10.1002/biot.201800062> (2018).

5 Salis, A. *et al.* Measurements and Theoretical Interpretation of Points of Zero Charge/Potential of BSA Protein. *Langmuir* **27**, 11597-11604, doi:10.1021/la2024605 (2011).

6 Sober, H. A. & Harte, R. A. *Handbook of Biochemistry (Selected Data Data for Molecular Biology)*. (CRC Press, 1973).

7 Yang, X. *et al.* Human Serum Albumin in the Presence of AGuIX Nanoagents: Structure Stabilisation without Direct Interaction. *Int J Mol Sci* **21**, doi:10.3390/ijms21134673 (2020).

8 Dobson, J. *et al.* Inducing protein aggregation by extensional flow. *Proceedings of the National Academy of Sciences* **114**, 4673-4678, doi:doi:10.1073/pnas.1702724114 (2017).

9 Furukawa, K. *et al.* Isoelectric point-amyloid formation of α-synuclein extends the generality of the solubility and supersaturation-limited mechanism. *Current Research in Structural Biology* **2**, 35-44, doi:<https://doi.org/10.1016/j.crstbi.2020.03.001> (2020).

10 Zhou, J. *et al.* Environmental Control of Amyloid Polymorphism by Modulation of Hydrodynamic Stress. *ACS Nano* **15**, 944-953, doi:10.1021/acsnano.0c07570 (2021).

11 Gasteiger, E. *et al.* in *The Proteomics Protocols Handbook* (ed John M. Walker) 571-607 (Humana Press, 2005).

12 Leuenberger, P. *et al.* Cell-wide analysis of protein thermal unfolding reveals determinants of thermostability. *Science* **355**, eaai7825, doi:doi:10.1126/science.aai7825 (2017).

13 Pettersen, E. F. *et al.* UCSF ChimeraX: Structure visualization for researchers, educators, and developers. *Protein Science* **30**, 70-82, doi:<https://doi.org/10.1002/pro.3943> (2021).

14 Kadri, U. *Long liquid slugs in stratied gas/liquid flow in horizontal and slightly inclined pipes*, TUDelft, (2009).

15 Movasaghi, Z., Rehman, S. & Rehman, I. U. Raman Spectroscopy of Biological Tissues. *Applied Spectroscopy Reviews* **42**, 493-541, doi:10.1080/05704920701551530 (2007).

16 Leulliot, N. *et al.* Unusual nucleotide conformations in GNRA and UNCG type tetraloop hairpins: Evidence from Raman markers assignments. *Nucleic Acids Research* **27**, 1398-1404, doi:10.1093/nar/27.5.1398 (1999).

17 Labesse, G. *et al.* Engineering, expression and biochemical characterization of the hemoglobin domain of a Erwinia chrysanthemi flavohemoprotein. *European Journal of Biochemistry* **253**, 751-759, doi:<https://doi.org/10.1046/j.1432-1327.1998.2530751.x> (1998).

18 Champion, P. M., Gunsalus, I. C. & Wagner, G. C. Resonance Raman investigations of cytochrome P450CAM from Pseudomonas putida. *Journal of the American Chemical Society* **100**, 3743-3751, doi:10.1021/ja00480a015 (1978).

19 Mołoń, M., Molestak, E., Kula-Maximenko, M., Grela, P. & Tchórzewski, M. Ribosomal Protein uL11 as a Regulator of Metabolic Circuits Related to Aging and Cell Cycle. *Cells* **9**, 1745 (2020).

20 Hui, C.-Y. & Jagota, A. Deformation near a liquid contact line on an elastic substrate. *Proceedings of the Royal Society A: Mathematical, Physical and Engineering Sciences* **470**, 20140085, doi:10.1098/rspa.2014.0085 (2014).

21 Zhu, C. *et al.* Characterizing hydrophobicity of amino acid side chains in a protein environment via measuring contact angle of a water nanodroplet on planar peptide network. *Proceedings of the National Academy of Sciences* **113**, 12946-12951, doi:doi:10.1073/pnas.1616138113 (2016).

22 Song, G. Bridging between material properties of proteins and the underlying molecular interactions. *PloS one* **16**, e0247147-e0247147, doi:10.1371/journal.pone.0247147 (2021).

23 Poirier, A., Banc, A., Stocco, A., In, M. & Ramos, L. Multistep building of a soft plant protein film at the air-water interface. *Journal of Colloid and Interface Science* **526**, 337-346, doi:<https://doi.org/10.1016/j.jcis.2018.04.087> (2018).

24 Chowdhury, S. R., Cao, J., He, Y. & Lu, H. P. Revealing Abrupt and Spontaneous Ruptures of Protein Native Structure under picoNewton Compressive Force Manipulation. *ACS Nano* **12**, 2448-2454, doi:10.1021/acsnano.7b07934 (2018).

25 Chowdhury, S. R. & Lu, H. P. Spontaneous Rupture and Entanglement of Human Neuronal Tau Protein Induced by Piconewton Compressive Force. *ACS Chemical Neuroscience* **10**, 4061-4067, doi:10.1021/acschemneuro.9b00295 (2019).

26 Shahu, L., Chowdhury, S. R. & Lu, H. P. Single-Molecule Human Nucleosome Spontaneously Ruptures under the Stress of Compressive Force: A New Perspective on Gene Stability and Epigenetic Pathways. *The Journal of Physical Chemistry B*, doi:10.1021/acs.jpcb.2c04449 (2022).
